# Ionization Engineering of Hydrogels Enables Highly Efficient Salt-Impeded Solar Evaporation and Night-Time Electricity Harvesting

**DOI:** 10.1007/s40820-023-01215-1

**Published:** 2023-11-06

**Authors:** Nan He, Haonan Wang, Haotian Zhang, Bo Jiang, Dawei Tang, Lin Li

**Affiliations:** School of Energy and Power Engineering, Key Laboratory of Ocean Energy Utilization and Energy Conservation of Ministry of Education, Dalian University of Technology, Dalian, 116024 People’s Republic of China

**Keywords:** Solar evaporation, Hydrogel evaporators, Salt impeding, Ionization engineering, Cyclic vapor-electricity generation

## Abstract

Interfacial solar evaporation holds immense potential for brine desalination with low carbon footprints and high energy utilization. Hydrogels, as a tunable material platform from the molecular level to the macroscopic scale, have been considered the most promising candidate for solar evaporation. However, the simultaneous achievement of high evaporation efficiency and satisfactory tolerance to salt ions in brine remains a challenging scientific bottleneck, restricting the widespread application. Herein, we report ionization engineering, which endows polymer chains of hydrogels with electronegativity for impeding salt ions and activating water molecules, fundamentally overcoming the hydrogel salt-impeded challenge and dramatically expediting water evaporating in brine. The sodium dodecyl benzene sulfonate-modified carbon black is chosen as the solar absorbers. The hydrogel reaches a ground-breaking evaporation rate of 2.9 kg m^−2^ h^−1^ in 20 wt% brine with 95.6% efficiency under one sun irradiation, surpassing most of the reported literature. More notably, such a hydrogel-based evaporator enables extracting clean water from oversaturated salt solutions and maintains durability under different high-strength deformation or a 15-day continuous operation. Meantime, on the basis of the cation selectivity induced by the electronegativity, we first propose an all-day system that evaporates during the day and generates salinity-gradient electricity using waste-evaporated brine at night, anticipating pioneer a new opportunity for all-day resource-generating systems in fields of freshwater and electricity.

**Supplementary Information:**

The online version contains supplementary material available at 10.1007/s40820-023-01215-1.

## Highlights

- An ionization-engineered hydrogel with electronegativity polymer chains to impede salt ions was designed.
- The hydrogel evaporator exhibited salt impedance in 20 wt% brine for 15 days with a high evaporation efficiency of 95.6%.
- An all-day high-salinity brine treatment with zero liquid discharge was proposed.

**Supplementary Information:**

The online version contains supplementary material available at 10.1007/s40820-023-01215-1.

## Graphical abstract

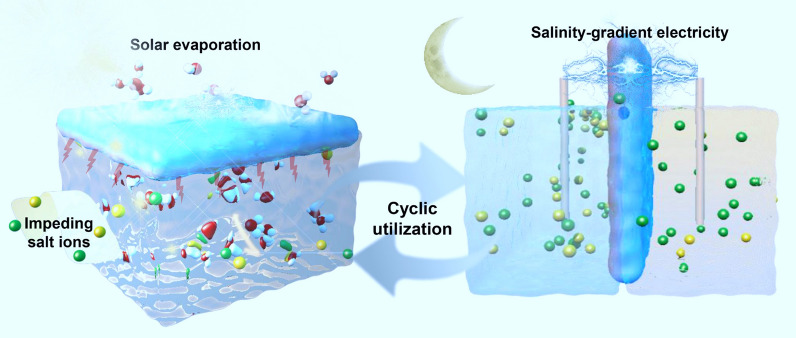

## Introduction

The freshwater crisis has emerged as a global systemic risk [1]. Seawater and brackish groundwater purification via thermal and membrane methods offer potential to bolster freshwater supply [2–6], yet the rapid proliferation of desalination plants raises quandaries. Fossil energy consumption and high-salinity brine discharge imperil marine ecosystems and human well-being [7–9]. Amidst this landscape, interfacial solar desalination garners significant attention. It outshines conventional methods in carbon emission reduction and high-salinity brine management (> 7.0 wt%) [10, 11]. However, attaining high-performance evaporators for high-salinity brine treatment remains demanding. Progressive materials offer promise in this endeavor, including wood-based derivatives like balsa wood, wood-derived aerogels, and Janus wood, which leverage natural interconnected channels to hinder salt fouling [12–14]. Representative instances include a 3D-printed tripodal porous composite evaporator mimicking wood's structure [15], a self-regenerating evaporator featuring artificial holes as salt-rejecting conduits [16], and silica nanofibrous aerogels inspired by reed leaves [17]. These innovations facilitate brine salt-free desalination, even at over 15 wt% concentration, producing water. Alternatively, hydrophilic porous media, like commercial sponges, electrospun fabrics, and nanofibrous aerogels, alleviate salt fouling via material adjustment and structural engineering [18–21]. Some fiber/fabric evaporators, exampled by polyaniline/halloysite decorated nanofiber composite evaporators, electrospun nanofibers, superhydrophilic polydopamine-modified carbon-fiber membranes, hierarchical photothermal fabrics, and vertically symmetrical evaporators based on photothermal fabrics, regulate wettability and manage structures to enable salt rejection [22–28]. Whereas, the above evaporators have several limitations so that their evaporation performance still has much room for improvement under one sun irradiation. Firstly, subject to the limited water transport capacity and water content of the common materials, the salt tolerance capacity, which is relying only on salt ions diffusion driven by a concentration gradient within the evaporator, is relatively poor. Secondly, the current salt-tolerant designs of common materials unavoidably damage the evaporation area or add additional vapor-escaping resistance; thus, it is hard to realize satisfactory high evaporation rates. Therefore, novel materials featuring high salt tolerance and highly efficient evaporation are desired for solar desalination in high-salinity brine.

Hydrogels, three-dimensional water-rich polymeric matrices, have emerged as a promising platform for efficient solar desalination, addressing the pressing need for high performance [29–31]. These salt-tolerant evaporators showcase unprecedented success, achieving a remarkable 4.0 kg m^−2^ h^−1^ evaporation rate in 3.5 wt% seawater, owing to their high water content and versatile physicochemical properties [32]. Specifically, benefiting from a water-rich environment, hydrogel-based evaporators possess excellent water transport and salt diffusion advantages [33–37]. Moreover, their tunable physicochemical properties allow for enhanced evaporation by altering the interaction between water molecules and polymer chains, expediting water activation [38, 39]. While these hydrogels have triumphed over common materials' limitations, their performance falters in brines exceeding 3.5 wt%. In these conditions, salt ions infiltrate the evaporator, escalating salt concentration, tarnishing light absorption, and compromising water supply, leading to diminished evaporation rates of as low as 1.3 kg m^−2^ h^−1^ [33, 34, 40]. The presence of mobile salt ions induces strong hydration, weakening hydrogel-water interaction, reducing water absorption, and increasing energy demands for evaporation. These challenges hinder efficient water replenishment and evaporation, impeding hydrogel-based evaporator advancement, necessitating scientific strides for effective high-salinity brine evaporation, and pushing forward this field to wide practical application.

**Fig. 1:**
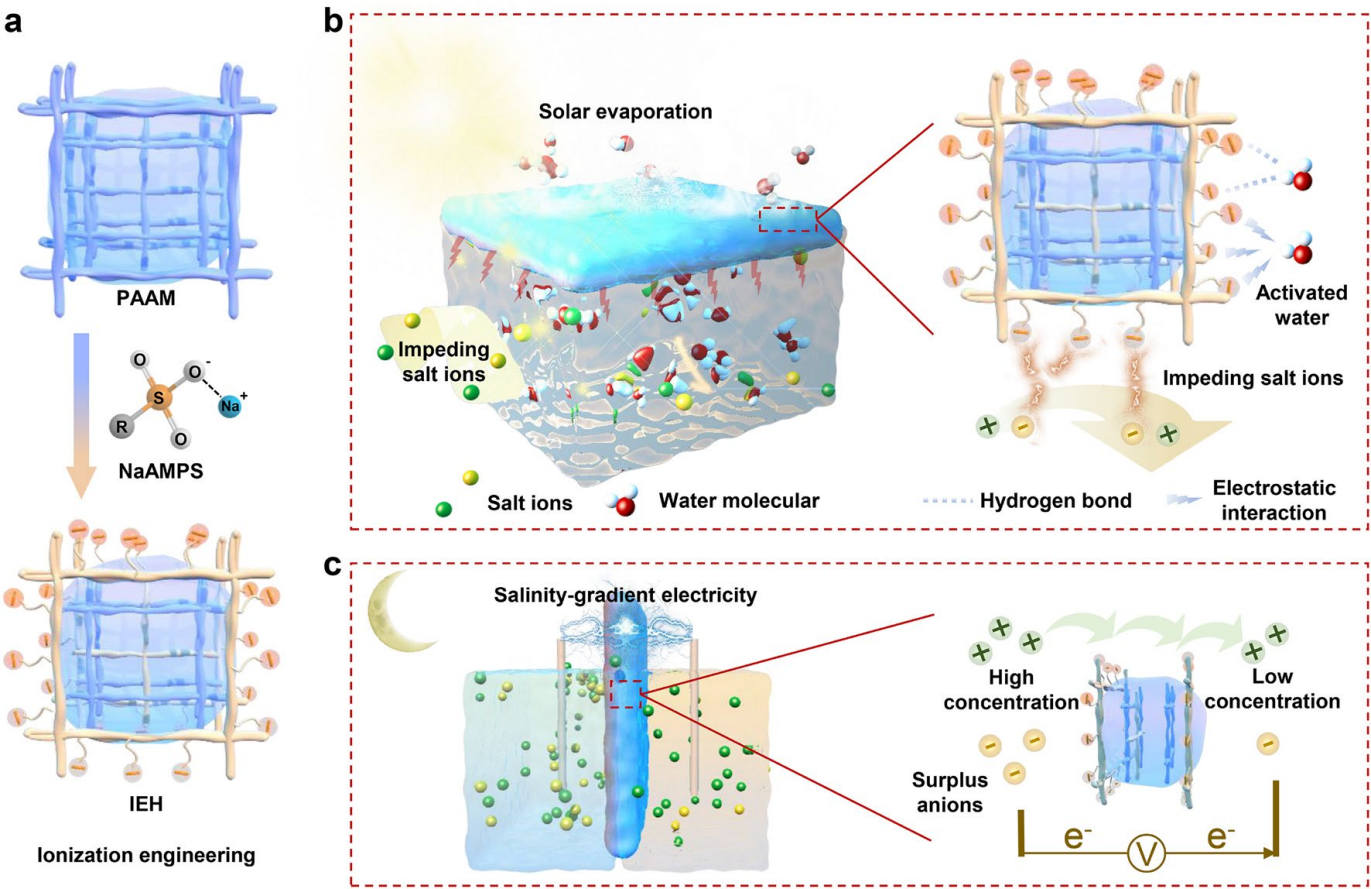
Schematic of ionization engineering for salt-impeded solar desalination during the day and generating electricity at night. **a** PAAM hydrogel networks containing water were ionization modified by NaAMPS, resulting in double networks, denoted as IEH, whose polymer chains feature electronegativity. **b** IEH loaded with SDBS-C suspends at the brine–air interface and converts solar energy to heat to generate vapor. In the process, the electronegative polymer chains impede salt ions and activate water molecules by hydrogen bonds and electrostatic interactions. **c** Salinity-gradient electricity generation at night. Under the cation selectivity of the IEH, cations diffuse from high to low concentration solution, while surplus anions are balanced via redox reactions based on the electrodes, thereby generating electricity

Here, we introduce ionization engineering of hydrogels to achieve simultaneous salt-impeded desalination and high-efficient evaporation. The ionization engineering is that the polyacrylamide (PAAM), an intrinsic network without ionizable groups, is interpenetrated by 2-acrylamido-2-methyl-1-propanesulfonic acid sodium (NaAMPS), which contains ionizable sulfonic acid groups (-SO_3_^−^). This forms the final polymerized hydrogel network, *i.e.*, ionization electronegativity hydrogel (IEH) (Fig. 1a). The presence of -SO_3_^−^ introduces fixed electronegative charges to the polymer chains in the hydrogel, thereby hindering anion permeability and achieving salt-impeded desalination. Simultaneously, these charges activate water molecules, promoting rapid evaporation. Sodium dodecylbenzene sulfonate-modified carbon black (SDBS-C) serves as the solar absorber. SDBS enhances carbon hydrophilicity, improving dispersion in sol solution. In water-rich hydrogels, SDBS-C boosts electronegativity, fine-tuning salt-impeding and water states via adjusting the hydrogen bonding network and intermolecular forces. In daylight, the IEH floats on the water–air interface, generating continuous vapor under one sun irradiation. Electronegativity impedes salt ions from entering evaporators through Coulomb repulsion, preventing salt fouling and enhancing salt tolerance. Combined electrostatic and hydrogen bond effects activate stored water for easy evaporation (Fig. 1b). Intermediate state water weakly interacts with polymer chains, bonding with fewer than four water molecules, an unstable condition [41], facilitating to break bonds during evaporation. The IEH solar evaporator achieves 2.9 kg m^−2^ h^−1^ evaporation rate and efficient salt impeding in 20 wt% brine, outperforming the current evaporators, even performing well (2.6 kg m^−2^ h^−1^) in deformed or extended (15-day) operations, comparable to many evaporators in seawater. Waste brine's salinity reduction and electricity generation are ingeniously managed. IEH's electronegativity selectively transports cations across a salinity gradient, while anions remain concentrated, initiating redox reactions for balanced solutions. This salinity gradient energy generates directional electron transport (Fig. 1c), harvesting energy without external power. Concurrently, waste brine's salinity meets re-evaporation standards. This study showcases ionization engineering's potential to surmount hydrogel evaporators' limitations, a crucial step in high-salinity brine desalination. The innovative strategy opens pathways for all-day systems: solar desalination during the day and salinity-gradient electricity generation at night, advancing water and energy solutions.

## Experimental Section

### Materials

Chemicals of IEH hydrogels including acrylamide (AAM), 2-arylamido-2-methyl-1-propanesulfonic acid sodium salt (NaAMPS) solution, N, N’-methylenebis (acrylamide) (MBAA), 2-hydroxy-4’-(2-hydroxyethoxy)-2-methylpropiophenone (UV-initiator), and agarose (AG) were purchased from Aladdin. Chemicals of the PVA pre-hydrogel consist of polyvinyl alcohol (PVA) with the average molecular weight of 15,000 (Aladdin), glutaraldehyde (50 wt% aqueous solution, Aladdin), and hydrochloric acid (HCl, 37%, Beijing chemical works, China). Chemicals of the PSA pre-hydrogel include sodium acrylate anhydrous (SA, 95%, Macklin, China) and ammonium persulfate (APS, AR, 98.5%, Macklin, China). Other chemicals include carbon black (Wanglin Biological co. LTD., Zhejiang, China), sodium dodecylbenzene sulfonate (SDBS, AR, Damao Chemical Reagent, Tianjin, China), sodium chloride (NaCl, AR, Tianjin Kemiou Chemical Reagent, China), potassium chloride (KCl, AR, Damao Chemical Reagent, Tianjin, China), and Copper (II) sulfate pentahydrate (CuSO_4_∙5H_2_O, 99%, Damao Chemical Reagent, Tianjin, China). The deionized (DI) water was from local sources. The self-suspension device was obtained by 3D printing based on ABS (acrylonitrile butadiene styrene) resin. The hydrogel shaping mold was a five by five centimeters quartz product. All materials were used without further purification.

### Preparation of Precursor Solution, Carbon Black, and IEHs

#### Preparation of the Precursor Solution

3 mol L^−1^ AAM (PAAM precursor solution) or 3 mol L^−1^ NaAMPS/AAM with an 8% molar ratio (NaAMPS precursor solution), 0.002 g MBAA, and 0.005 g UV-initiator were added into DI water to form the two precursor solutions. 2 wt% AG concerning the weight of the solution was added as a cross-linker for sequential polymerization. The solution was heated at 60 °C for 25 min on a magnetic stirrer at 300 rpm to obtain a transparent solution with 8 mL.

#### Pre-treatment of the Carbon Black

Carbon black is a common solar absorber in interfacial evaporation. Enhancing its solubility, we used 0.3 wt% sodium dodecyl benzene sulfonate (SDBS) for pretreatment, followed by triple DI water washes to yield SDBS-modified carbon black (SDBS-C).

#### Preparation of the IEHs

For the fabrication of the IEHs with different contents of NaAMPS, the above two hot precursor solutions were sealed into different pre-prepared quartz products at volume ratios of 1:0, 3:1, 1:1, 1:3 and 0:1 and then, cooled at 25 °C for 30 min, ensuring pre-gelation. The obtained two pre-hydrogels were placed in contact and were irradiated with 365 nm ultraviolet light under 45 W irradiation power for 2 h to obtain IEH0, IEH1, IEH2, IEH3 and IEH4, in which the NaAMPS was 0, 2, 4, 6, and 8 mol%. In the irradiation process, the PAAM pre-hydrogel with 0.03 g SDBS-C added was placed on the bottom to form a photothermal IEH.

#### Preparation of the PVA Precursor Solution

0.8 g of PVA particles with the alcoholysis of 87–89 mol% was dissolved in 8 mL of DI water with continuous stirring for 30 min at 25 °C. 85 μL of glutaraldehyde (50% in H_2_O), 0.005 g UV initiator, and 800 μL HCl (3 wt%) were added through bath sonication. Then, the hybrid sol was poured into the custom-made mold. The gelation lasted for 4 h at 70 °C [38].

#### Preparation of the PSA Precursor Solution

The SA, MBAA and APS were uniformly mixed and dissolved in deionized water (8 mL) at a molar ratio of 50:1:1 to make a homogeneous solution. Similarly, the hybrid sol was poured into the custom-made mold. The gelation lasted for 4 h at 70 °C [42].

#### Preparation of the PVA-NaAMPS Hydrogel and PAAM-SA Hydrogel

The PVA-NaAMPS hydrogel and the PAAM-SA hydrogel were synthesized using the same process as that for the IEHs. The SDBS-C was added in the PVA solution and the PAAM solution, respectively.

### Characterizations

Scanning electron microscopy (SEM, ZEISS Gemini SEM 300) measurements were performed to observe the cross-sectional morphology. The cryo-SEM images were taken by a biocryo-scanning electron microscopy (Regulus8220). The elemental distributions of sodium, sulfur, carbon and chloride were analyzed using energy-dispersive X-ray spectroscopy (EDS) mapping in SEM. The Fourier transform infrared (FTIR) spectra were conducted by a FTIR spectrometer (Thermo Scientific Nicolet iS5) with a DTGS KBr detector and a beam splitter using the KBr substrate material. The Raman spectra were characterized by a spectrometer (Renishaw invia, LabRam HR Evolution). The Raman mapping was conducted in the same Raman spectrometer. The excitation radiation for the Raman emission was produced using a laser with a laser wavelength of 532 nm. The X-ray photoelectron spectra (XPS) were measured by an X-ray photoelectron spectroscopy (Thermo Scientific, USA) equipped with an excitation source of Al-Kα X-ray. The mechanical properties were evaluated by compression and stretch tests using an electronic universal testing machine (CMT6103, MTS). The heat flow in water phase change in the IEHs was measured by a differential scanning calorimetry (DSC, TA Q200, USA). The swollen water contents were measured by an electronic balance (Mettler Toledo ME55). The salinity of NaCl solutions was detected by a salinity refractometer (DR201). The IR images were captured by an IR camera (FLK-T1480P). The concentration of chloride ions was measured by an ion chromatograph (ICS5000). The sodium ion concentration was evaluated by an inductively coupled plasma source mass spectrometer (Agilent 7700X & Agilent 7800). The photothermal temperature response was tracked by a K-type thermocouple with a data acquisition (DAQ) board (Agilent 34970A, USA). The UV–vis-NIR spectra measurement was performed by a UV–vis-NIR spectrometer (Daojin UV-3600). The electrical signals were monitored by a multimeter (Keithley 6510). The Zeta potential was measured by a nanoparticle size and zeta potential analyzer (DLS) (Malvern Zetasizer Nano ZS90). Both FTIR spectra and Raman spectra characterizing chemical composition were analyzed with KnowItAll Academic Edition software, which is a completely free set of chemical software packages provided by Wiley for academia.

### Solar Evaporation Test

Conducted at the Key Laboratory of Ocean Energy Utilization and Energy Conservation of Ministry of Education, Dalian University of Technology, the experiment employed a class AAA solar simulator (71S0503A, Sofn, China). This simulator delivered a consistent solar flux of 1000 W m^−2^ (equivalent to one sun irradiation) within a controlled laboratory setting at 25 °C and 60% relative humidity. Optical density was measured using an optical power meter (S310C, Thorlabs, USA) positioned at the same level as the absorber. Temperature data were captured using a K-type thermocouple connected to an Agilent 34970A data acquisition (DAQ) board (USA). Monitoring of brine mass loss due to evaporation was executed with a PTX-FA 210s electronic balance (China). As reported previously [43], the absorber-to-beaker lip and absorber-to-beaker wall distances were minimized, while the absorber-to-light source distance was extended. This configuration facilitated unhindered vapor escape during evaporation. Prior to activating the solar simulator, a 10-min waiting period ensured complete wetting of the evaporator by bulk brine.

## Results and Discussion

### Chemical Composition of the IEH

**Fig. 2:**
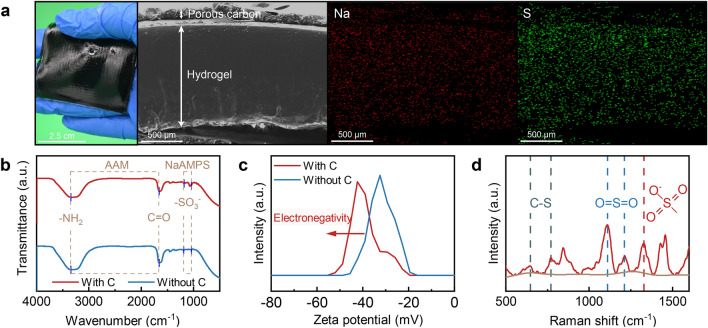
Chemical composition of the IEH. **a** Photograph of a photothermal IEH. Cross-sectional SEM image of the dehydrated IEH without containing water. Corresponding EDS mapping of sodium in red and chloride in green. **b–c** FTIR spectra and Zeta potential of IEHs with and without SDBS-C. **d** Raman spectrum of IEH

To fabricate the electronegative hydrogel, a sequential network formation method and a contact polymerization method were employed. The contact polymerization confines the SDBS-C within the side of the hydrogel, illustrated as Fig. S1a, localizing heat on the photothermal evaporation. The fundamental procedure commences by generating a transparent ionized PNaAMPS hydrogel and a PAAM hydrogel with SDBS-C deposited at the bottom. These two hydrogels are contacted to polymerization under ultraviolet radiation to obtain the final hydrogel network. As SDBS-C remains exclusively in the PAAM hydrogel, the resulting evaporator possesses SDBS-C only on one side. In addition, Fig. S1b demonstrates that the SDBS-C enables a high absorption of 95.2% across the whole spectra of solar irradiation, including UV–vis. Thus, the SDBS-C on the bottom functions as a strong ultraviolet absorber to bring the NaAMPS monomer uniformly diffusing and polymerization (Fig. S2). Figure S4 showcases cryo-SEM images of the hydrogel at different resolution, effectively demonstrating its porosity and hydrophilic structure. The pore diameter, ranging from several nanometers to micrometers, ensures the water transport and vapor escape. Additionally, the interlinked hydrogel channels slightly change with the addition of carbon black and different monomer ratios (Fig. S5). SEM cross-sectional observation of the IEH presents a dense structure of the hydrogel layer and a carbon layer (Fig. 2a). Elemental mapping of sodium and sulfur demonstrates uniform distribution of ionized NaAMPS within the IEH. Such ionization engineering is also a generalized regulation strategy for hydrogels, endowing most of hydrogels with electronegativity to break the hydrogel bottleneck of impeding salt. On the basis of this method, we successfully designed PVA-NaAMPS and PAAM-SA hydrogels using PVA and SA (sodium acrylate) as replacements for PAAM and NaAMPS, respectively, opening the door to this material application in solar salt-impeded desalination (Fig. S6).

FTIR spectra, revealing the chemical composition of the IEH, are depicted in Fig. 2b. In the spectrum of the pure IEH without SDBS-C, the peaks at 3345 and 1659 cm^−1^ correspond to the amidogen and amide carbonyl stretching vibration, the characteristic peaks of AAM. The NaAMPS exhibits distinctive absorption bands at 1183 and 1043 cm^−1^, which are due to the symmetric and asymmetric vibration of the –SO_3_^−^ group. The signal peak of the –SO_3_^−^ group in the IEH with SDBS-C added is stronger than that without SDBS-C. The phenomenon is attributed to the pre-treatment of carbon with SDBS, acting as a surfactant. The SDBS also ionizes and produces –SO_3_^−^. The combined effect of NaAMPS and SDBS makes the IEH exhibit a high electronegativity (Figs. 2c and S7). Additionally, the Raman spectrum, complementary to the XPS, was used to further investigate the chemical composition. The Raman spectrum in Fig. 2d reveals peaks at 649 and 770 cm^−1^ corresponding to C–S stretching. Peaks at 1110 and 1213 cm^−1^ represent the symmetric stretching of S=O bonds, while the signal at 1328 cm^−1^ is attributed to R-SO_3_^−^. The XPS survey scan spectrum shows N_1s_, S_2p_, and Na_1s_ peaks attributed to AAM and NaAMPS (Fig. S8a) [44]. Spectra of S_2p_ and Na_1s_ are assigned to sulfonate and sodium in the NaAMPS and SDBS-C (Fig. S8c, d) [45]. The results above consistently confirm that the presence of sulfonate in the IEH. Compared with polar groups such as carboxyl and hydroxyl, the sulfonic acid group is a strong polar group that is easily ionized and is charged negatively in a water-rich environment [45, 46]. In the IEH, NaAMPS and SDBS-C generate sulfonic acid groups and sodium ions, in which sulfonic acid groups of NaAMPS endows the polymer network with electronegativity, while sodium ions are confined by the polymer network and are in a free state in the network.

### Water Activation, Salt Impeded, and Evaporation Characteristics Based on IEHs

**Fig. 3:**
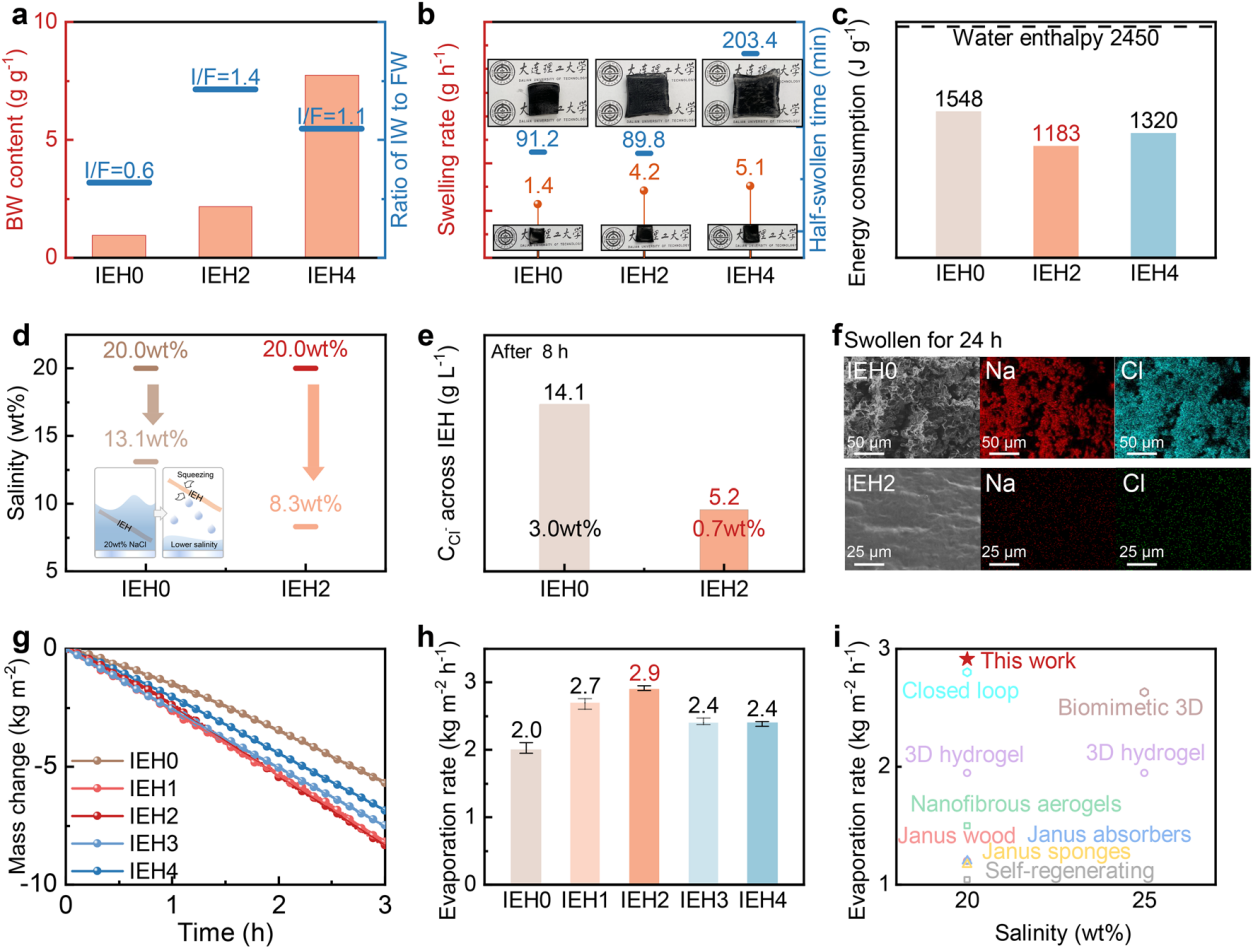
Water activation, salt impeded, and evaporation characteristics based on IEHs. **a** Enhanced BW content and related IW/FW ratios from the IEH0 to the IEH4. **b** Swelling rates and half-swollen time increasing from IEH0 to IEH4. **c** Energy consumption of IEHs in evaporation. Vaporization enthalpy of pure bulk water is 2450 J g^−1^. **d** Schematic diagram and data of conscious-permeable and squeezing salt-impeded tests based on IEHs. **e** Concentration of chloride ions permeated across IEHs in 20 wt% brine after 8 h. **f** SEM images and EDS mapping of IEHs after being immersed in 20 wt% brine for 24 h. **g–h** The fastest mass change and mean evaporation rates of three parallel experiments in 20 wt% brine with IEHs under one sun irradiation. Slight fluctuations in the data are shown in the form of error bars. **i** Comparison in evaporation rates in 20 wt% and 25 wt% brine of the IEH2 and the reported literature

It is precise because of the electronegativity of polymer chains and SDBS-C, the water states in the IEH change obviously. On the basis of the intensity of water-chains interaction, water contained in the IEH is divided into free water (FW), intermediate water (IW), and bound water (BW). IW has been demonstrated as activated water that requires less energy to evaporate and FW makes the greatest contribution to water transport (Fig. S9a) [47]. Raman spectra confirm the existence of the two water states in the IEH (Fig. S9b). In particular, the water states are able to be improved by electronegativity, which is induced by both SDBS-C and the polymer chains, and the individual hydrophilicity. This is because electronegative atoms in hydrophilic functional groups effectively capture water molecules through the strong attraction, such as hydrogen bonds or electrostatic forces [30, 48]. The interaction facilitates the adjustment of the hydrogen bonding network and intermolecular forces, thus tuning the proportion of free water, intermediate water, and bound water. The proportion of high IW/FW ratio is optimized and improved toward facilitating evaporation. SDBS-C contributes by activating intermediate water with a high IW/FW (Fig. S9e) and reducing evaporation enthalpy (Fig. S9f). To systematically assess the influence of the ionization degree of polymer chains on the water states, we fabricated IEHs with the same SDBS-C content and different content of NaAMPS, denoted as IEH0, IEH1, IEH2, IEH3, and IEH4 (Fig. S10). NaAMPS content is 0, 2, 4, 6, and 8 mol% of the monomer, respectively. IEH0, featuring 0 mol% NaAMPS, represents the pure PAAM hydrogel with only SDBS-C addition. The specific analysis was conducted by IEH0, IEH2 and IEH4. As indicated by Figs. 3a and S11, increased ionization increases BW content, which strongly interacts with polymer chains. Apparently, the ability of the IEH2 and the IEH4 to capture water molecules is enhanced because of the ionization. The IW/FW ratios for both IEH2 and IEH4 exceed that of IEH0, highlighting that ionization engineering effectively triggers water activation within hydrogels. Whereas, excessive ionization in IEH4 leads to a higher FW content than IW. This is because the electronegativity-induced ionization makes the polymer chains repel each other, thereby providing much space to accommodate FW. In sum, after ionization, IEH2 presents the highest IW/FW ratio, while IEH4 possesses the highest BW content.

Variations in water content and corresponding water states within the IEHs have a significant influence on both water transport and evaporation. Concerning water transport, the time-dependent water content (*Q*_s_) (Fig. S12) and swelling rates of the IEHs (Fig. 3b) rise with the ionization. This trend stems from the propensity of sulfonic acid groups in polymer chains to attract water molecules. Related photographs displaying fully swollen and dry IEHs are shown in Fig. 3b insets. Among these, IEH4 presents the highest swelling rate, while the time required for fully swollen is the longest compared with other samples. This observation, aligning with the trend in BW content, corroborates that ionization significantly enhances water absorption and transport. Same results were from water contact angle measurements (Fig. S13). Concerning water evaporation, the evaporation energy consumption (*E*_ec_) of IEHs is lower than the enthalpy of bulk water (Fig. S14). This discrepancy is attributed to the confinement of water within hydrogel nanochannels and molecular meshes, effectively reducing the energy demands for evaporation [49]. Through controlled dark experiments, IEH2 and IEH4 require low E_ec_ (Figs. 3c and S15), evidencing that ionization further reduces the energy demands for evaporation. Among them, the IEH2 reaches the lowest E_ec_, suggesting that the highest ratio of IW to FW in IEH2 is beneficial to evaporation. Because the IW, which has weak interactions with polymer chains, is in an unstable state, demanding less energy for evaporation compared to FW [41]. While IEH4 showcases remarkable water transport capacity, the significant contained water restricts the energy utilization, necessitating higher energy for evaporation than IEH2. Consequently, IEH2 stands out for its exceptional water absorption ability and the lowest evaporation energy consumption.

Salt tolerance is a crucial factor in determining whether the evaporation performance is considerable in brine. IEH0 and IEH2 were performed in following analyses to investigate the significance of ionization engineering in impeding salt ions. Firstly, a simple conscious-permeability experiment was conducted (see details in Section S1.12 in Supporting Information). After swollen in 20 wt% brine, IEH2 yielded 8.3 wt% light brine, while IEH0 generated 13.1 wt% brine, underscoring IEH2's effective salt-impeding capability (Fig. 3d). To further prove the salt-impeded performance, we employed Raman mapping and osmotic swelling test to directly assess the water absorption capacity of IEH2 in brine. In contrast to its initial state, the IEH2 displays a substantial color change within five minutes of swelling, indicative of complete water molecule occupation (Fig. S16a). Even in saturated brine, IEH2 still maintains the water absorption ability (Fig. S16b) [50]. Secondly, the ability of the IEHs to impede chloride ions under 20 wt% brine conditions was accurately evaluated (see details in Section S1.13 in Supporting Information). Given sodium ion presence in IEH2, chloride ions were chosen as representatives of salt ions. Depicted in Fig. 3e, after eight hours, the chloride ion concentration passing across IEH2 is nearly half of that of IEH0, indicating the effectiveness of the ionization engineering for impeding salt. Chloride ion concentration across the other IEHs data is shown in Fig. S17. SEM images and EDS mapping of dried IEHs swollen in 20 wt% brine for 24 h (Fig. 3f) provided more conclusive evidence. Agglomerated salt crystals destructively invade the interior of the IEH0, leading to significant deformation and obvious collapse. In contrast, the IEH2 contains only a small amount of salt ions, and its morphology remains intact. Apparently, ionization engineering makes the polymer chains negatively charged with the fixed sulfonic acid groups. According to the Gibbs–Donnan theory [51, 52], this electronegativity impedes salt anions from entering the evaporator. Meanwhile, cations are also impeded to ensure electroneutrality (see details in Section S1.13 in Supporting Information). Notably, the enhancement in electronegativity stemming from the introduced SDBS-C also contributes to the promotion of salt-impeding properties (Fig. S18). In addition, the electronegativity is affirmed its efficacy in impeding negatively charged impurities (Fig. S19). Therefore, the IEH2 is promising for highly efficient salt-impeded desalination in high-salinity brine.

Demonstrated in Fig. 3g, h, the evaporation rates based on IEHs in 20 wt% brine under one sun irradiation are 2.0, 2.7, 2.9, 2.4, and 2.4 kg m^−2^ h^−1^, respectively. Each experiment was conducted with three parallel measurements under identical conditions. IEH2 presents the highest evaporation rate of 2.9 kg m^−2^ h^−1^ with 95.6% efficiency (Fig. S20), which is a high value in relation to other reported literature (Fig. 3i) [13, 16, 19, 34, 53–56]. The remarkably high evaporation rate is attributed to the reduced evaporation enthalpy (Fig. 3c), resulting from the high IW/FW ratio (Fig. 3a) triggered by the presence of hydrophilic sulfonic acid groups. In addition, we also observed a decrease in evaporation enthalpy with increasing salt concentration from 3.5 to 20 wt% (Fig. S21) [39].

### Solar Desalination Performance in 20 wt% Brine Under One Sun Irradiation

**Fig. 4:**
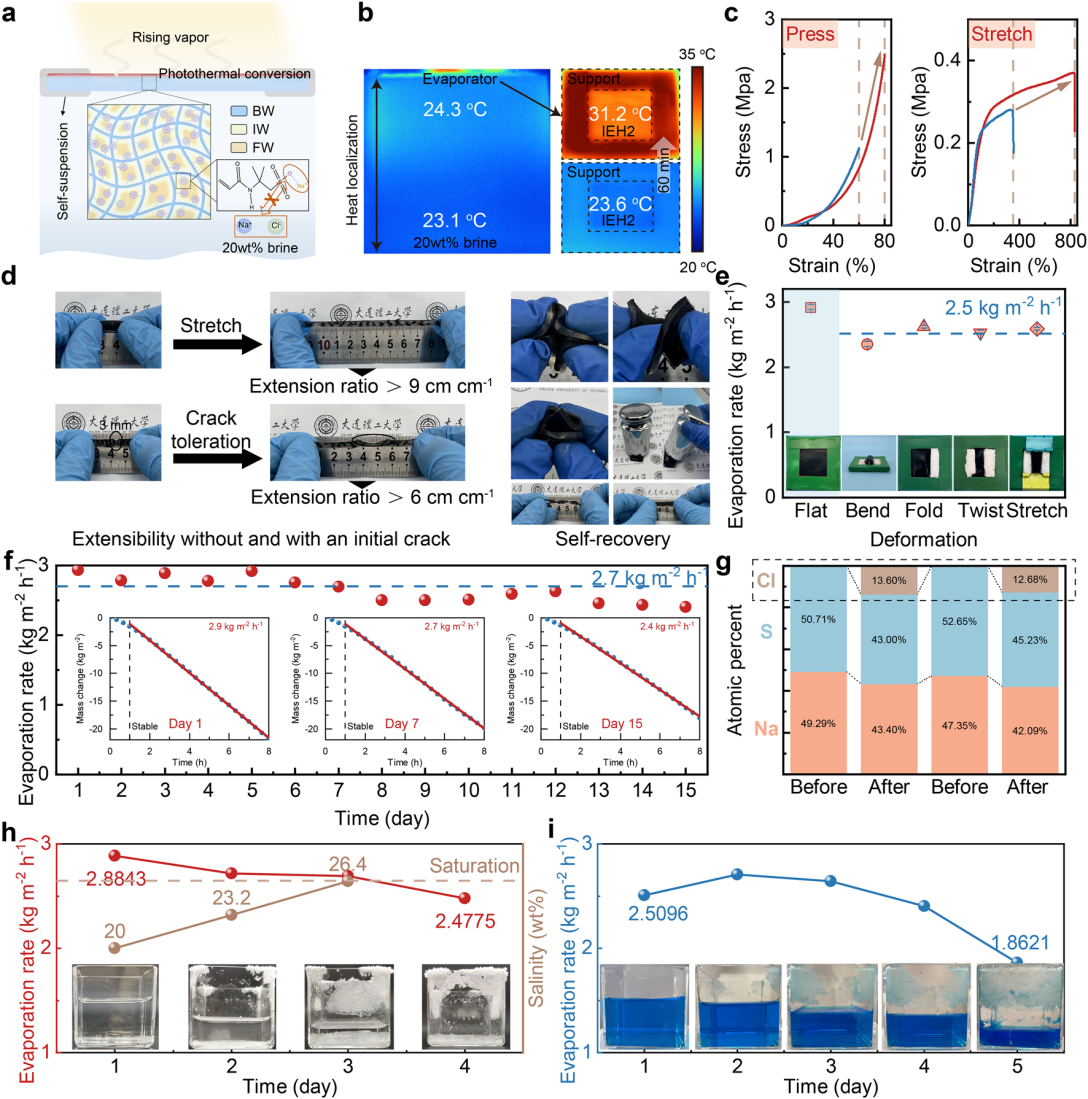
Solar desalination performance in 20 wt% solutions under one sun irradiation. **a** Schematic illustration of the IEH2 suspending on the brine surface. **b** Infrared images showing the temperature distribution on the device side surface in the steady-state and the temperature response on the evaporation surface. **c** Compression and stretching properties before and after ionization, which are shown in blue and red, respectively. **d** Photographs of the extensibility of the IEH2 and self-recovery behavior of the IEH2 under highly folded and twisted. **e** Evaporation rates and corresponding photographs of the IEH2 under different deformation. **f** Evaporation rates of the IEH2 in 20 wt% brine for 15 days under one sun irradiation. Insets are typical mass change behaviors for 1 day, 7 days, and 15 days. **g** Two parallel measurements of elemental semi-quantitative analysis showing the amount of sodium, chloride, and sulfur ions in the IEH2 before and after 15-day solar desalination. **h–i** Evaporation rates over time with initial 20 wt% NaCl and 20 wt% CuSO_4_ solutions until water and solute are entirely separated. Insets are the time-dependent solution photographs

To further test the limit in the solar desalination capabilities of the IEH2, we conducted solar evaporation experiments in 20 wt% brine under one sun irradiation, subjecting it to diverse and challenging conditions. Throughout these experiments, the IEH, supported by self-suspension, was positioned at the brine–air interface (Fig. 4a), wherein only the upper surface was exposed to generate vapor, ensuring a continuous water supply. The water interacting with the polymer chains includes BW, IW, and FW. The ionization-induced electronegativity impedes salt ions. Infrared images confirm the equilibrium heat localization effect and temperature response. Within 60 min, there is a temperature rise of 7.4 °C, subsequently stabilizing at a uniform surface temperature of 31.2 °C (Fig. 4b). Meantime, most heat is confined at the water–air interface, facilitating evaporation. The temperature of the self-suspension support exceeds that of the IEH2, indicating the high-performance evaporation takes a lot of heat from the IEH2.

Deformation resistance and durability are pivotal evaluation metrics in solar desalination. Initially, we analyzed the mechanical properties of IEH2 (Fig. 4c). The double-network IEH2 consisting of PAAM and PNaAMPS exhibits impressive mechanical resilience, which is not obvious in the single-network PAAM hydrogel [57]. As the compressive strain approaches 80%, IEH2 withstands a stress of 2.49 MPa, signifying substantial compressive resistance. Conversely, the single-network PAAM hydrogel only endures a compressive strength of 1.13 MPa with a 60% strain. Besides, the IEH2 shows outstanding stretch ability. Compared with the tensile property of the single-network PAAM hydrogel, the strain of the IEH2 increases by 479%, high strength rises by 0.09 MPa, and elasticity modulus grows by 0.02 MPa. Deformation-resistant photographs of IEH2 are shown in Fig. 4d, showcasing stretching up to nearly nine times its initial length. Even for a notched sample with a 3-mm crack, it is stretched to six times of the original length, demonstrating the capacity to disperse energy focused at the crack during stretching [58]. The resilience of the IEH2 was further evaluated by highly intensive folding and the samples fully recovered their original state after unloading. Subsequent experiments involve bending, folding, twisting, and stretching IEH2 in 20 wt% brine (Fig. 4e). The accompanying photographs depict varying degrees of deformation. Remarkably, the average evaporation rate is stable at 2.5 kg m^−2^ h^−1^. The wholescale resilient assessment of the IEH2 proves its long-term adaptability to complex environments in practical applications.

A continuous eight-hour one sun irradiation measurement in 20 wt% brine was performed for half a month, measuring daily evaporation rates (Fig. 4f). IEH2 consistently maintains evaporation rates at 2.7 kg m^−2^ h^−1^, highlighting its long-term stability and endurance for solar desalination without any maintenance. Typical evaporation behaviors on day 1, day 7 and day 15 all maintain stability, respectively. After the 15-day operation, Fig. S23 demonstrates that the press capability has almost no attenuation, while the stretch capability weakens but still retains about 400% strain, meeting the need for floating on the water surface to evaporate. Elemental semi-quantitative analysis (Fig. 4g) indicates minimal Cl^−^ infiltration in IEH2. Comparing the stability data of IEH1, IEH2, and IEH3 (Fig. S24) emphasizes that enhanced evaporation stability aligns with improved salt impeding performance. It indicates that ionization engineering endows evaporators with impeding salt. The impeding capability makes it more difficult for salt ions to enter the evaporator, but it is truly difficult to completely block salt ions because the evaporator was in contact with the 20 wt% brine all the time in the experiment. In short, all results above strongly indicate that the IEH2 is resistant to different complex conditions and the extreme evaporation rates rank among the best values in the reported literature (Fig. S25), quite reliable for practical long-term solar desalination.

Can the evaporation rates and desalination performance remain satisfactory when the salinity of 20 wt% solutions increases further? These were verified by treating NaCl and CuSO_4_ solutions. The results show that the IEH2 enables solar desalination until the complete separation of water and solute (Fig. 4h, i). Illustrated in the photographs, as water progressively evaporates from the solution, the solution level descends, leading to corresponding increases in salinity until only solute remains once water has fully evaporated. The remaining solute could be easily recycled and reused, showing the great potential for zero leftover brine and minimum additional energy consumption. After a four-day desalination process resulting in oversaturated brine, only 3.9 wt% Cl^−^ is detected in the evaporator, which is well below the salt accumulation threshold and ensures the evaporator remains unimpeded. Pitifully, although the IEH2 exhibits effective evaporation during the entire process, the evaporation rates drop slightly because of the supersaturated solution.

### Night-time Salinity-gradient Electricity Generation Performance

**Fig. 5:**
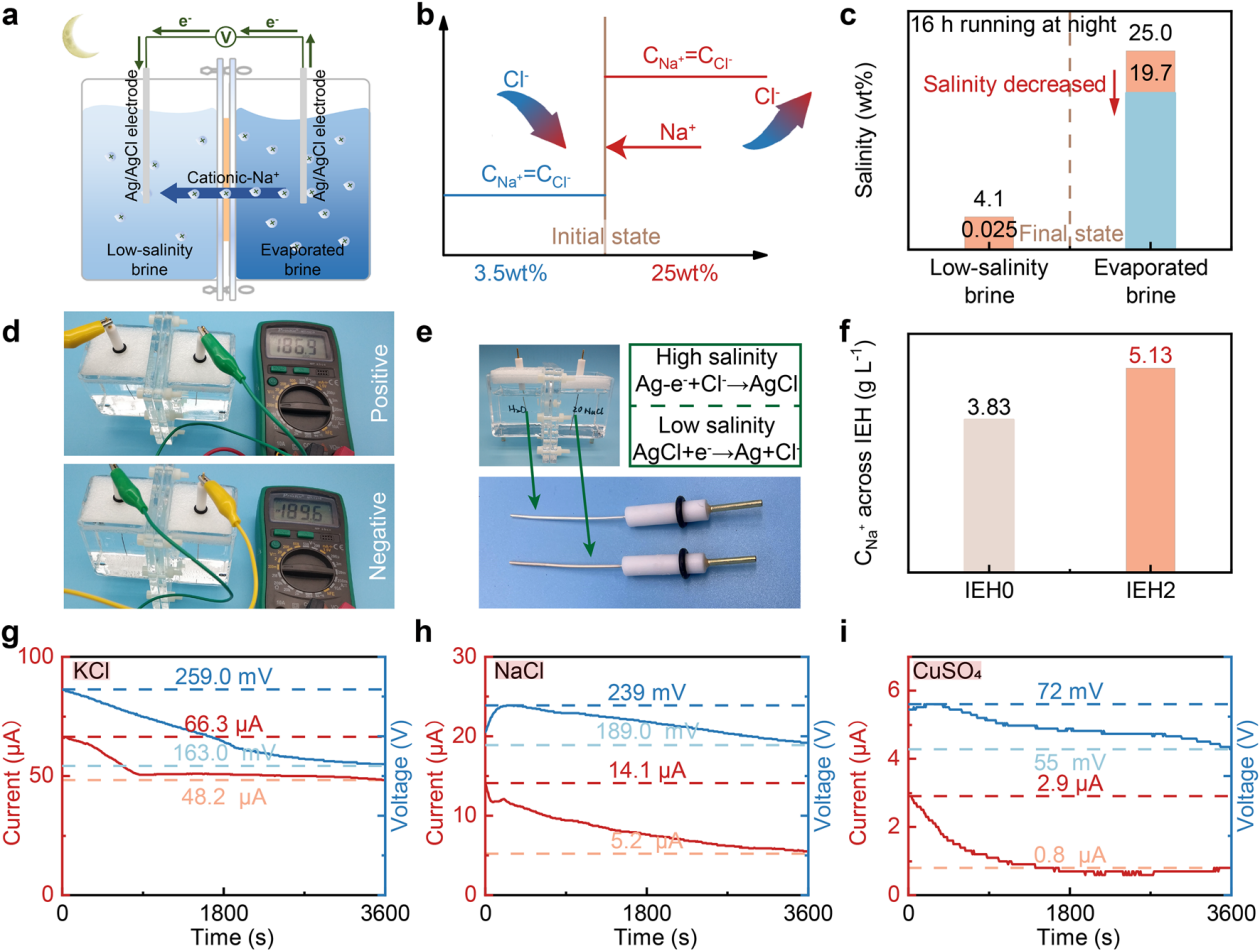
Night-time salinity-gradient electricity generation performance. **a–b** Schematic of the waste-evaporated brine recycling and the salinity-gradient electricity generation setup. **c** After 16 h running at night, the salinity of the waste evaporated brine decreased to less than 20 wt%. **d** Test photographs showing the direction of the voltage and cation-ion flow. **e** The redox reaction equations of electrodes and the corresponding electrode photographs. **f** Concentration of sodium ions after 8 h permeation experiments. **g–i** Measured current and voltage curves of the IEH2 in KCl, NaCl, and CuSO_4_ conditions

To address the aforementioned concerns, we ingeniously designed a night-time recycling system that balances the salinity of day-time evaporated brine and generates salinity-gradient electricity for night-time convenience. On the basis of the inherent advantages of hydrogels, IEH2 is able to function as nanofluidic channels for ion flow [59, 60]. Its electronegativity selectively facilitates cation transport while repelling anions, enabling gradient energy conversion [61, 62]. Figure 5a, b depicts a typical experimental setup and schematic diagram. IEH2 is positioned between high-salinity evaporated brine and low-salinity brine. Under the salinity gradient, cations are preferentially transported across the IEH2 toward the low-salinity side. This cation-preferred ion diffusion leads to a charge separation process, resulting in an electrochemical potential difference between the two electrodes, leading to the generation of voltage. The Ag/AgCl electrodes undergo a redox reaction in response to the electrochemical potential difference, thereby recording current. Meantime, after 16-h running at night, the salinity of waste-evaporated brine decreases from 25.0 to 19.7 wt%, reaching the standard for high-performance re-evaporation during the daytime (Fig. 5c). Evidently, cation selectivity is pivotal to the entire system, as the voltage and current arise from ion separation [63]. To confirm the cation-selectivity behavior of the IEH2, we assessed the potential direction. Figure 5d shows that electron flow direction aligns with the net cation flow from high to low salinity. Reversing the multimeter's electrode probes results in potential reversal. Similar results were observed in two electrodes, where the one on the high-salinity side turns gray because of the loss of electrons from Ag, while the other on the low-salinity side gradually whitens because of the electrons gained by Ag^+^ (Fig. 5e). AgCl on the anode (losing electrons) forms as AgCl on the cathode (receiving electrons) gets consumed. Thus, considering the entire system, the electrodes are not entirely consumed. Accordingly, the electricity is obtained upon the selective cations of Na^+^ over Cl^−^ from high to low salinity across the IEH2. Importantly, Fig. 3e demonstrates the anion-impeded property of the IEH2 and Fig. 5f verifies the cation-preferred permeable selectivity of the IEH2, compared with the IEH0. Therefore, the sulfonic groups in the IEH2 function as cations attractors and anions repellents, generating an effective electricity performance.

To demonstrate the broad application potential, we explored three different configurations of solutes in waste-evaporated solutions, including KCl, NaCl, and CuSO_4_, within the system. The corresponding electrical outputs are recorded in Fig. 5g–i. Under a salinity gradient, which is defined as *C*_high_/*C*_low_ = 1000, the diffusion current and voltage are of the same polarity, mainly attributed to the cation permselectivity. However, the current and voltage values during the same period are very different. The reason is that different salt ions have different migration capabilities, which are governed by factors including the ionic valence state and ionic hydration [64]. Cu^2+^ has a larger ionic valence (z = 2) than Na^+^ and K^+^ (z = 1), which reduces electromotive force and weakens ion transport and charge separation. As a result, the current and voltage levels are the lowest [65, 66]. The highest performance of KCl is due to the poor hydration capability of K^+^. Compared with Na^+^ and Cu^2+^, K^+^ binds fewer water molecules in brine, resulting in a smaller hydrated shell and, eventually, smaller ionic size and lower ion transport resistance [67, 68]. It should note that the IEH is also capable of generating electricity using low salt gradient between the 25 and 3.5 wt%, which is equivalent to seawater, to highlight the practicality (Fig. S26). Significantly, the obtained voltage surpasses 50 mV, representing an advancement beyond prior reports [69, 70]. The continuous operation is demonstrated by the *in situ* outdoor experiment of day-time solar-driven evaporation for eight hours and 16-h electricity generation (Fig. S27). Furthermore, compared with previously reported evaporators that generate vapor and electricity simultaneously [69], this approach enables a mutually beneficial relationship between electricity generation and evaporation. It utilizes the salinity gradient between bulk brine and low-salinity brine, such as seawater. The higher electricity performance is attributed to the higher salinity of the waste brine, without compromising the evaporation. Instead, the high-efficiency salt-impeded evaporator would leave over high-salinity bulk brine, conducive to electricity generation. Therefore, our system provides advantages in both energy harvesting and resource utilization during the night compared to most evaporators that remain idle at night.

## Conclusions

In conclusion, our developed IEH successfully achieved highly salt-impeded solar desalination during the day and salinity-electricity conversion at night because of the ionization-induced electronegativity. We demonstrate that ionization engineering establishes electrostatic interaction with water molecules, resulting in active water and lowered the energy requirement for efficient evaporating. Furthermore, the electronegativity effectively impedes salt ions in high-salinity brine. The obtained IEH enables a high solar evaporation rate of 2.9 kg m^−2^ h^−1^ with 95.6% efficiency under one sun irradiation. Moreover, the IEH exhibits high mechanical stability and resistance against challenging environments, including high deformation, a 15-day ultra-long period, oversaturated brine, and nearly saturated heavy metal wastewater. These attributes highlight its significant potential for practical solar desalination applications. Meantime, under the function of cation selectivity, the IEH balances the salinity of the waste-evaporated brine and offers salinity gradient electricity at night. This work provides an effective approach to ionization engineering of hydrogels, endowing the polymer chains with uniform electronegativity and anticipating substantial advances in the production of clean water during the day and electricity at night, well poised for long sea-voyage and remote islands deployment.

## Supplementary Information

Below is the link to the electronic supplementary material.

Supplementary file1 (PDF 2069 KB)
