# Supplementary material for: Ionization Engineering of Hydrogels Enables Highly Efficient Salt-Impeded Solar Evaporation and Night-Time Electricity Harvesting
Source: Nanomicro Lett. 2023 Nov 6;16:8. doi: 10.1007/s40820-023-01215-1 (PMC10628017; doi:10.1007/s40820-023-01215-1)
Supplement: Supplementary file 1 — Supplementary file1 (PDF 2069 KB) [file 40820_2023_1215_MOESM1_ESM.pdf]

Supporting Information for

Ionization Engineering of Hydrogels Enables Highly Efficient Salt-Impeded Solar Evaporation and Night-Time Electricity Harvesting

Nan He[1], Haonan Wang[1], Haotian Zhang[1], Bo Jiang[1], Dawei Tang[1], Lin Li[1, *]

[1]School of Energy and Power Engineering, Key Laboratory of Ocean Energy Utilization and Energy Conservation of Ministry of Education, Dalian University of Technology, Dalian 116024, P. R. China

*Corresponding author. E-mail: lilinnd@dlut.edu.cn (Lin Li)

S1 Supplementary Figures and Tables

S1.1 Schematic and Characterizations of Photothermal Properties

The hydrogel evaporator with side-loaded carbon has a thermal conductivity of 0.51 W m^{-1} K^{-1} at around 30 °C. The carbon absorbs solar radiation and converts it to heat through lattice vibration [S1, S2]. The absorption (A) is 95.2%, which is calculated by $A = 1 - T - R$ (Fig. S1b). The stable temperature of the dried IEH with side-loaded absorbers rapidly rises within six minutes to reach a steady 50°C (Fig. S1c), which was 5.3°C higher than that with uniform-loaded absorbers. The tested sample undergoes one sun irradiation without the contact to water.

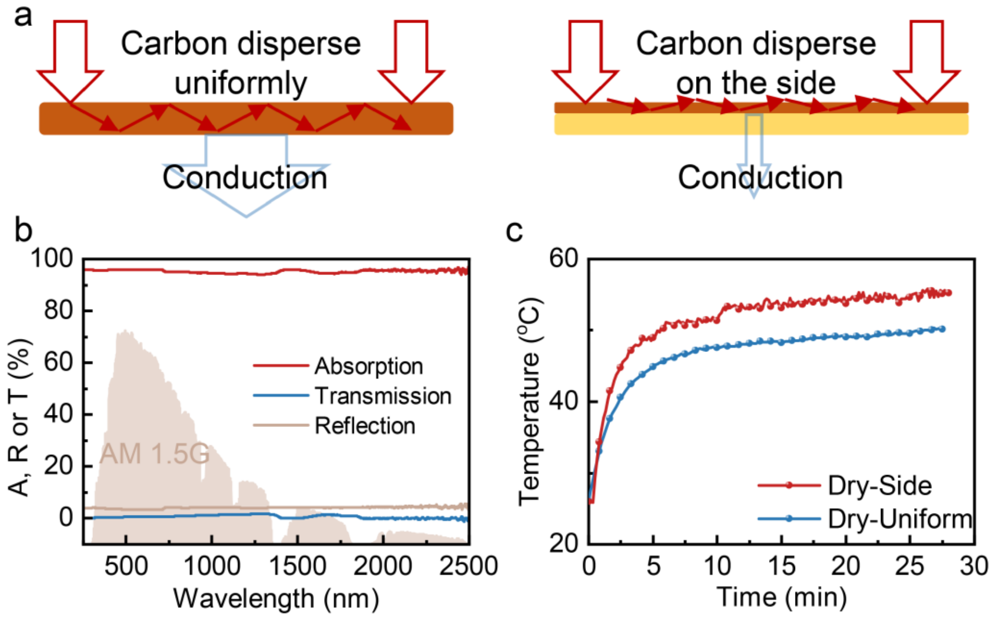

Fig. S1 a The left is the schematics of carbon (Red) disperse uniformly inside the hydrogel. The right is the schematic of carbon disperse on the side of the hydrogel (Yellow), of which the heat conduction is reduced. **b** Reflection (R) and transmission (T) of the IEH with carbon. **c** Photothermal conversion of the dried IEH, which undergoes one sun irradiation without the contact to water

S1.2 Characterizations of the Hydrogel with and without SDBS-C

According to the phenomenon in the experiment, carbon may enable ionized NaAMPS uniformly distributed in hydrogels. To verified this, we placed a carbon paper on the bottom of the hydrogel during the material synthesis. For a hydrogel without carbon paper, the ionized

NaAMPS monomer only diffused in a micrometer range (Fig. S2a, right). The NaAMPS monomer uniformly diffuses in a millimeter range, and a charge-uniform hydrogel was obtained (Fig. S2b). The possible reason can be summarized as follows: The polymerization process involves ultraviolet light crosslinking. Carbon has a high absorption capacity within the ultraviolet range (Fig. S1b). As a result, during the polymerization, carbon facilitates the concentration of ultraviolet light onto the sample, leading to increased absorption by the ultraviolet photo-initiator [S3, S4]. This enhanced absorption excites the molecules within the hydrogel, contributing to the uniform filling of NaAMPS in the sample.

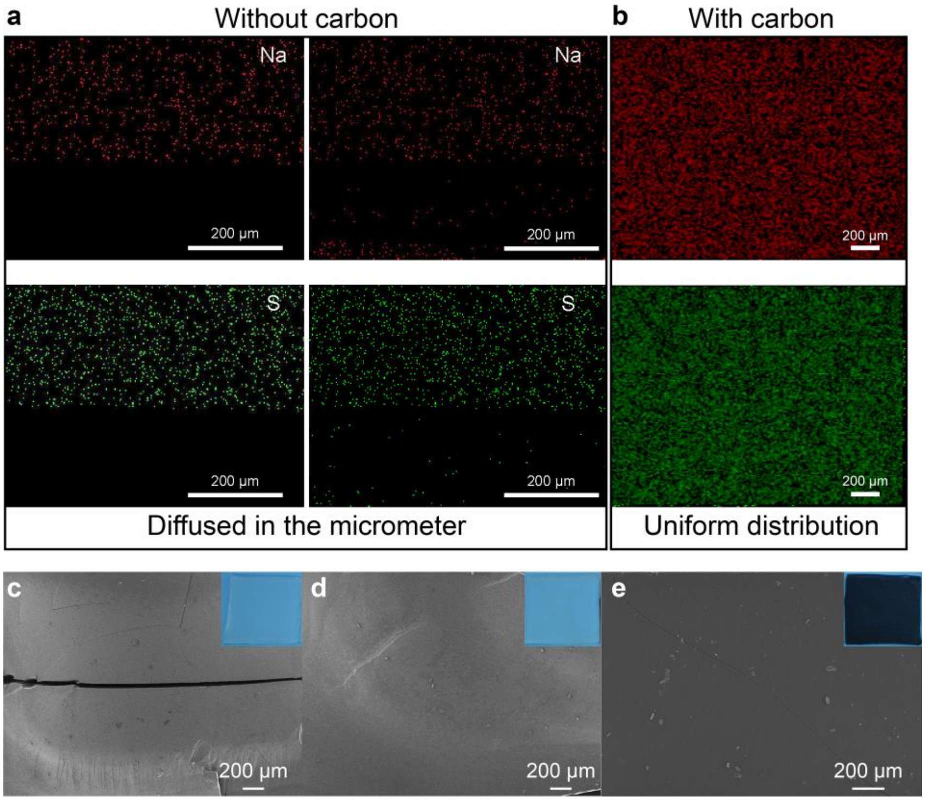

Fig. S2 a EDS mapping of the hydrogel without carbon. The left and right are the distribution of the characteristic elements before and after diffused polymerization, respectively. **b** EDS mapping of the hydrogel with carbon. **c** SEM and sample image of the hydrogel without carbon before polymerization. **d** SEM and sample image of the hydrogel without carbon after polymerization. **e** SEM and sample image of the hydrogel with carbon

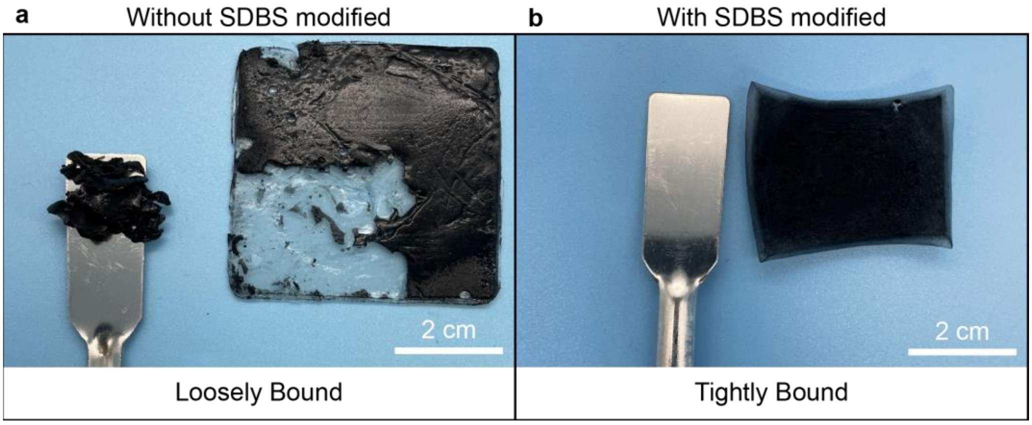

Fig. S3 a–b Photos of the carbon in hydrogels with and without SDBS modified

S1.3 Cryo-SEM Images of Internal Structure

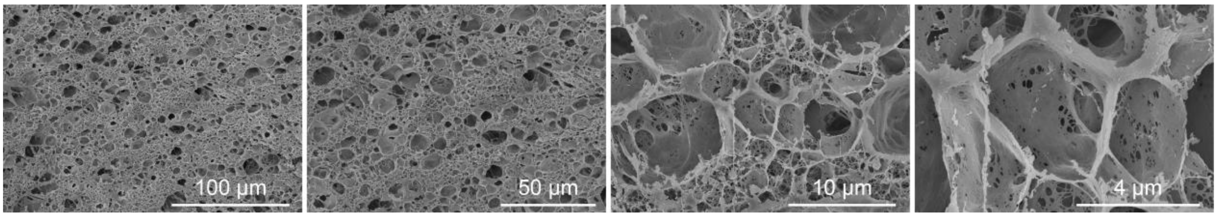

Fig. S4 Cryo-SEM images of the IEH with SDBS-C at different resolution

Comparing IEHs with (Fig. S5a) and without SDBS-C (Fig. S5b) reveals that the absence of carbon results in narrower interlinked hydrogel channels. Additionally, contrasting the IEH (Fig. S5b) with an IEH featuring a higher proportion of ionized monomers (Fig. S5c) demonstrates that increased ionized monomer content leads to an enlargement of interlinked hydrogel channels.

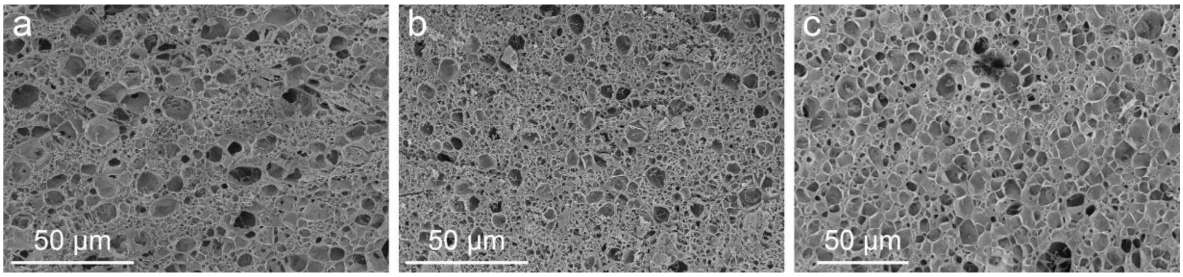

Fig. S5 Cryo-SEM images of **(a)** IEH with SDBS-C addition, **(b)** IEH without SDBS-C addition, and **(c)** IEH without SDBS-C and containing a high proportion of ionized monomers

S1.4 Demonstration of Generalization of Ionization Engineering

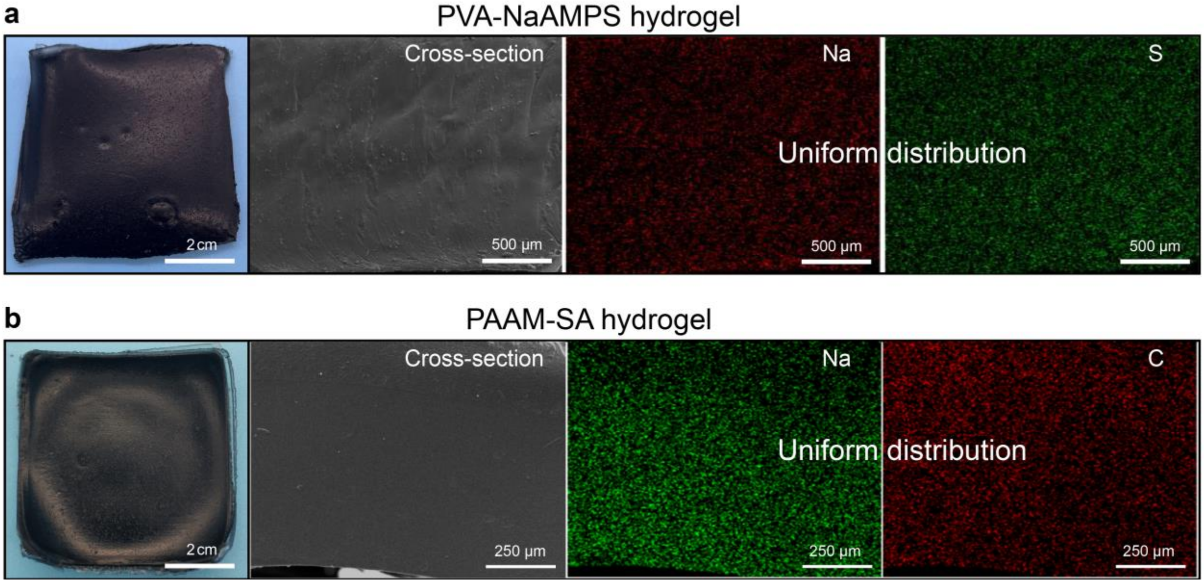

Fig. S6 a–b Characteristic elements of NaAMPS and SA are uniformly distributed in the cross-section of PVA and PAAM hydrogels

S1.5 Zeta Potential of IEH0

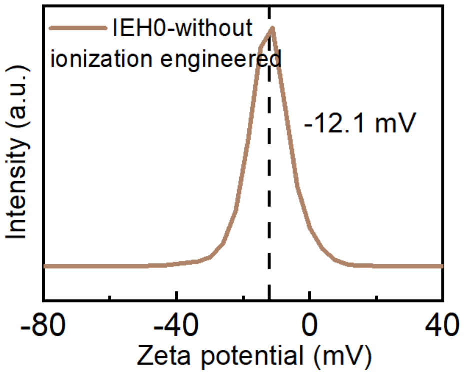

Fig. S7 Zeta potential of the IEH0 without ionization engineering under pH=7.0. The value is -12.1 mV

S1.6 XPS Spectra of the IEH

The IEH has the typical peaks of C_{1s}, N_{1s}, Na_{1s} and S_{2p} at 285.33, 399.97, 1071.8, and 168.42 eV (Fig. S8a). All spectra were calibrated based on the binding energy of C–C/C–N, *i.e.*, 284.8 eV. The two peaks of N_{1s} located at 399.6 and 400.9 eV were attributed to the N–H bond and the C–N bond (Fig. S8b). The existence of S_{2p} and Na_{1s} represents the inclusion of NaAMPS in the PAAM hydrogel (Fig. S8c–d). In C_{1s} spectra, the peaks at 284.8, 286, 287.4, and 288.8 eV represent C–C, C–O/C–N/C–S, C=O, and O=C–O (Fig. S8e). Note that there are no O=C–O bonds in AAM and NaAMPS, but the UV-initiator has these bonds, thus they were detected [5].

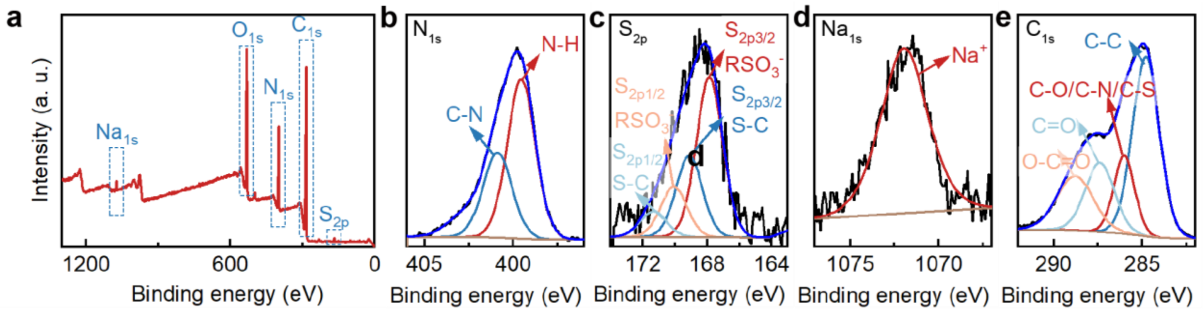

Fig. S8 a XPS survey scan spectrum and high-resolution spectra of **b** N_{1s}, **c** S_{2p}, **d** Na_{1s}, and **e** C_{1s}

S1.7 Water States in the IEH

The water molecules with weakly hydrogen-bonded interaction and with non-hydrogen-bonded with polymer chains, which are named as IW and FW (Fig. S9a), can be measured. Raman spectra were fitted by two peaks through Gaussian function (Fig. S9b) [S6]. Among them, the peak at 3408 cm^{-1} in a red fitting curve is IW and the peak at 3203 cm^{-1} in a blue fitting curve is FW.

S1.8 Investigation of the SDBS-C in Tuning Water States and Evaporation Enthalpy

We conducted measurements of the melting behavior and evaporation capability of water in IEHs using a differential scanning calorimeter (DSC). For the melting measurement, the IEH was carefully sealed in an Al crucible to prevent water evaporation, and the entire sample was frozen at -30 °C for 20 min to ensure complete freezing. The experiment was then carried out with scans at a linear heating rate of 2 °C min^{-1} in the temperature range of -30 to 30 °C, under a nitrogen flow flux of 50 mL min^{-1}.

As depicted in Fig. S9c–d, the frozen water in hydrogels with or without carbon was all easy to melt than normal water. Also, for water in hydrogels, the melting enthalpy was much lower than that of normal water. These results indicate that the water in the hydrogels exhibits different

thermodynamic behaviors from the normal bulk water, and its intermolecular potential is low. For evaporation, a high ratio of intermediate water to free water (IW/FW) can reduce evaporation enthalpy and facilitate evaporating [S7, S8]. Based on the Fig. S9d, we evaluated the IW/FW of hydrogels with and without carbon. The pink peak is related to intermediate water melting, and the light blue peak represents free water melting. The IW/FW of the IEH with SDBS-C is 1.4, which is slightly higher than the IEH without SDBS-C (Fig. S9e).

During the evaporation capability measurement [6, 9], the IEH was closely sealed in an Al crucible and the entire sample was heated from 30 to 200 °C with the increase rate of 5 °C min^{-1} under a nitrogen flow flux of 20 mL min^{-1}. Fig. S9f indicates that the IEH with SDBS-C enables low heat energy consumption.

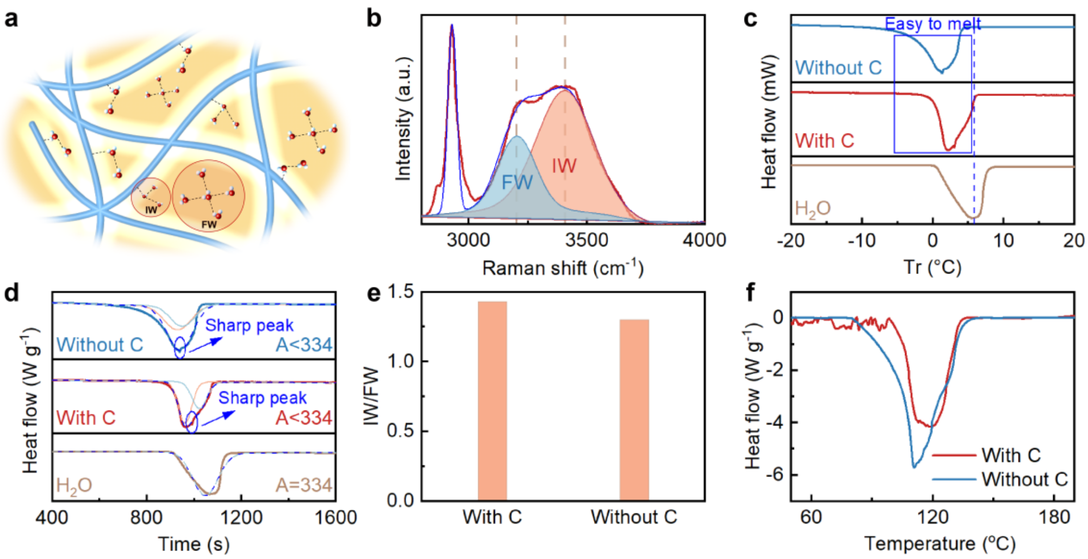

Fig. S9 **a** Diagrammatic illustration of water in the polymer networks. **b** Raman spectra corresponding fitting curves of the swollen IEH2. **c–d** DSC curves of the melting behavior of frozen water in IEHs and normal water. Among fitting curves and peaks, pink represents IW, while blue expresses FW. **e–f** IW/FW ratios and DSC curves of the evaporation behavior of the IEH2 with and without SDBS-C

S1.9 Investigation of Ionized Monomers in Tuning Water States and Evaporation Enthalpy

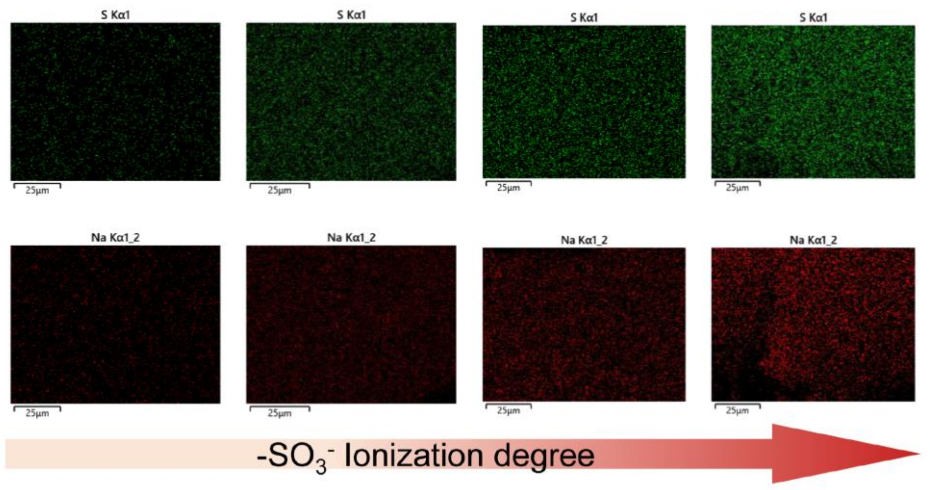

Fig. S10 EDS mapping of IEH1, IEH2, IEH3 and IEH4 from left to right, showing the increasing ionization degree and the characteristic elements are sodium and sulfur

DSC melting curves are shown in Fig. S11a, b. Both Fig. S11a and b originate from the same

dataset, with only the difference in horizontal coordinates. The peak separation treatment has been conducted and presented in Fig. S11a [S8], in which fitting curves and found peaks of IW and FW were displayed in pink and blue, respectively.

The bound water is unfrozen water, thus there are no peaks for BW during the measurement [S10]. The BW content was calculated by:

$$W_{BW} = W_s - W_{IW} - W_{FW} \tag{S1}$$

where W_s is the total water content in the saturated IEH, W_{IW} and W_{FW} are the content of intermediate water and free water, respectively. W_{IW} and W_{FW} can be estimated by the following Eqs. (2) and (3):

$$W_{IW} = \Delta H_{IW} W_s / \Delta H_m \tag{S2}$$

$$W_{FW} = \Delta H_{FW} W_s / \Delta H_m \tag{S3}$$

where ΔH_{IW} and ΔH_{FW} are the melting enthalpy of IW and FW from DSC measurement. ΔH_m is the saturated melting enthalpy of normal water, 334 J g^{-1}.[11] W_s is represented by [7]:

$$W_s = (M_s - M_d) / M_d \tag{S4}$$

where M_s and M_d are the mass of the fully swollen sample and corresponding dried samples. Note that the mass of the fully swollen IEH was tracked using a balance until the value was not changed. The two-hour data are shown in Fig. S12. The calculated content of the three water states in IEHs is listed in Table S1.

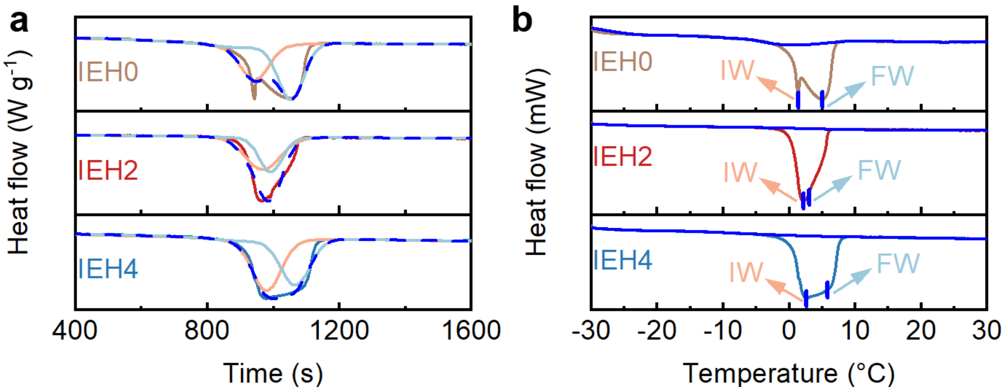

Fig. S11 a–b DSC curves of the melting behavior of frozen water in IEHs

Table S1 Content of three types of water and corresponding melting enthalpy of fully swollen IEHs

	W_s	ΔH_{IW}	ΔH_{FW}	W_{IW}	W_{FW}	W_{BW}	IW/FW
IEH0	4.16	100.00	157.00	1.24	1.95	0.96	0.64
IEH2	12.68	163.00	114.00	6.19	4.33	2.16	1.43
IEH4	34.88	136.00	124.00	14.20	12.95	7.73	1.10

S1.10 Measurement of Water Transport Performance of IEHs

Based on the swelling experiments, the water transport was measured [12]. All IEHs to be tested were completely dried in the heated oven. Then, the dried samples were immersed in pure water at the room temperature of 25°C and a humidity of 60%. The water mass in these swollen IEHs denoted as Q_s was recorded by a balance for 5 min until their mass was no change in 30 min. The results are shown in Fig. S12. Note that the IEH4 was fully swollen in 270 min. Given that IEHs are nearly fully swollen during the solar evaporation process, the swelling rate from a

half-saturated state to a fully-swollen state can represent the water transport of the IEH in the evaporation process. The swelling rate is calculated by:

$$v_{swelling} = 0.5W_s \ / \ t_{0.5} \tag{S5}$$

where $t_{0.5}$ is the time from a half-swollen state to a fully-swollen state. The W_s, $t_{0.5}$, and calculated $v_{swelling}$ are shown in Table S2.

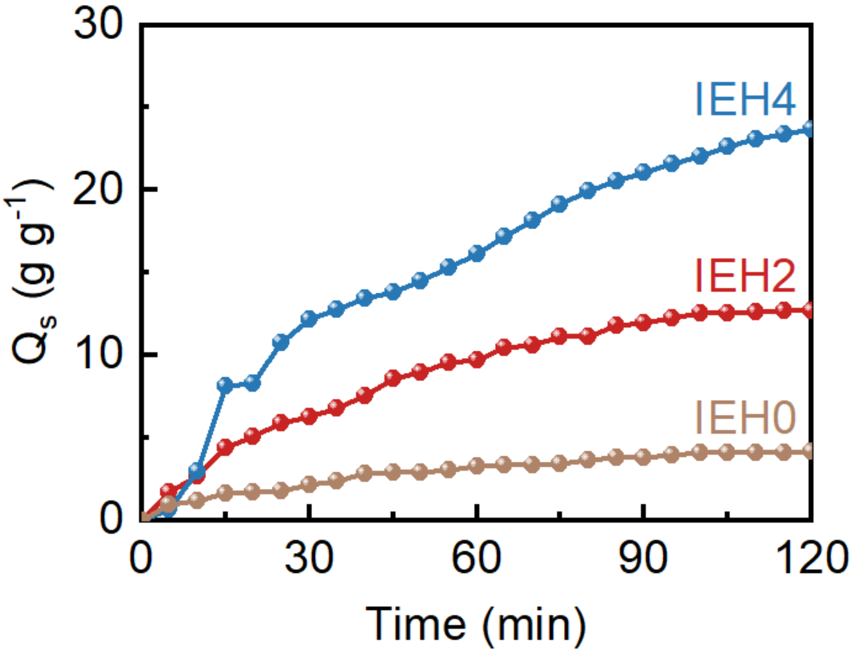

Fig. S12 Water content per gram of dried samples overtime in the swelling process

Table S2 Water content, swelling time, and swelling rates of IEHs from a half-swollen state to a fully-swollen state

	IEH0	IEH2	IEH4
$0.5W_s$	2.08	6.34	17.44
$t_{0.5}$ (min)	91.20	89.80	203.40
$v_{swelling}$ (g h^{-1})	1.37	4.24	5.14

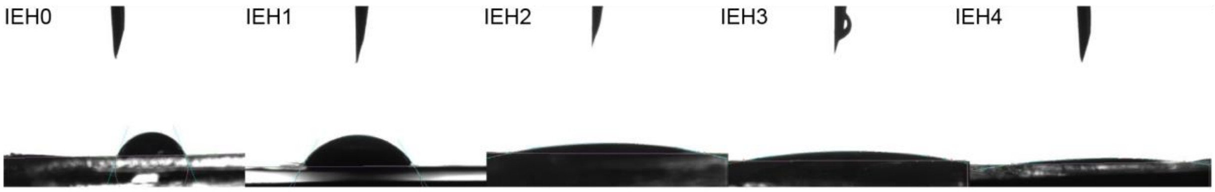

Fig. S13 Water contact angles of IEHs in the same testing time

S1.11 Estimation of Energy Consumption with IEHs

DSC measurements in Fig. S14 show the heat flow changes with temperature increasing and with time going on. It indicates that normal bulk water evaporated immediately when the temperature was high enough, and the corresponding enthalpy was calculated to 2580 J g^{-1}, which is closed to 2450 J g^{-1}. The heat flow curve of the IEH2 was broad and change gradually, confirming its different evaporation behavior from bulk water. The related enthalpy was measured to 1420 J g^{-1}. The value is much lower than that of bulk water.

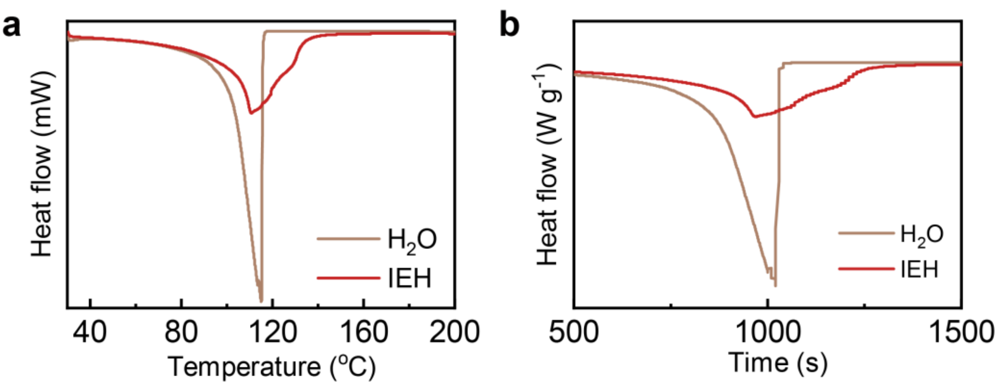

Fig. S14 a–b DSC curves of the evaporation behavior of the IEH2 and normal bulk water

Controlled dark experiments were conducted to estimate the energy consumption of the IEHs during the evaporation experimen [S6]. In this control experiment, the evaporation area of brine was equal to that of the IEH. Both experiments were carried out simultaneously in a closed container under the same room temperature of 25 °C and relative humidity of 60%, ensuring the same energy input (U_{in}). The energy consumption (E_{ec}) in Fig. 3c was estimated using the following equation:

$$U_{in} = E_0 m_0 = E_{ec} m_g \tag{S6}$$

where U_{in} is the identical power input (1 kW m^{-2} h^{-1}), E_0 and m_0 are evaporation enthalpy (2450 J g^{-1}) [S13] and mass change of pure water without the evaporator, m_g is a mass change rate of IEHs under the same experimental condition (Fig. S15). Note that the measured enthalpy of the IEH is lower than DSC results, this is because water evaporation completely during the DSC test and slightly dehydration in the evaporation process [S6].

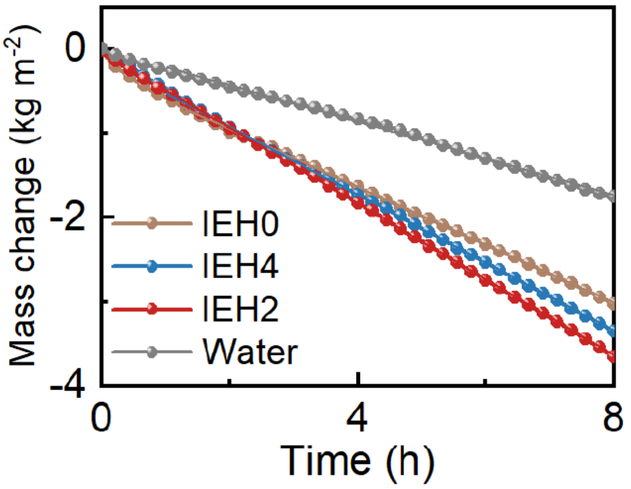

Fig. S15 Mass change of water in IEHs under dark conditions

S1.12 Conscious-permeability Experiment

The conscious-permeability experiment was improved according to the previous report [S14]. Firstly, the IEHs were thoroughly dried in a heated oven. Next, the dried IEHs were immersed in 20 wt% brine for 48 h. Due to the osmotic pressure difference between the IEH and the surrounding brine, water molecules and free ions entered the dried IEHs, causing them to swell completely and form a hydrogel containing a large amount of brine. Subsequently, the excess surface brine from the IEHs was removed and discarded using absorbent filter paper. Finally, the fully-swollen IEHs were mechanically squeezed, and the salinity of the obtained brine was measured using a salinity refractometer.

S1.13 Water Absorption and Salt-impeded Capacity of the IEH2 in Brine

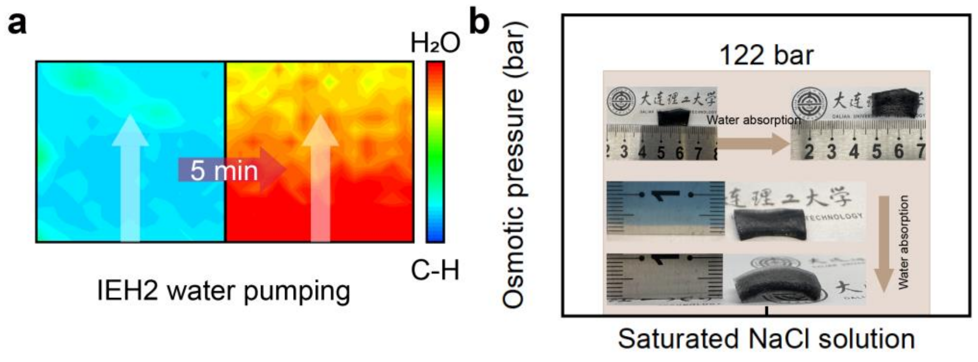

Fig. S16 a Raman mapping of the IEH2 in a dried state and after absorbing water for 5 min in 20wt% brine. **b** Photos of the IEH2 before and after absorbing water in saturated brine that has 122 bar osmotic pressure

Experimental procedure for Cl- concentration across the IEH:

Figures S17, 3e, and 5f were obtained using the electrical setup, which is shown in Fig. 5a. The DI water and 20 wt% brine was on the different sides of the IEH2. Driven by the salinity difference, salt ions diffused from brine to DI water. After a period, an appropriate amount of the solution in the original DI water side was taken and measured the concentration of ions.

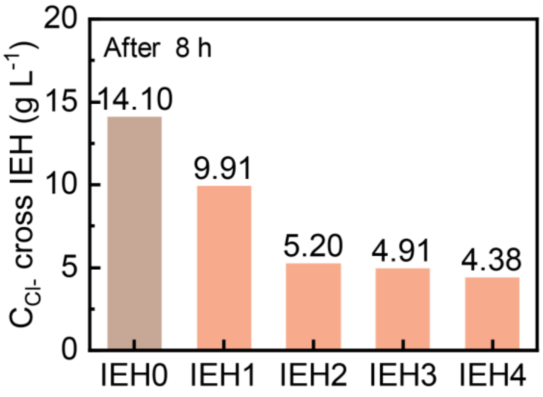

Fig. S17 Concentration of chloride ions permeated across IEHs in 20wt% brine after 8 h

Detailed salt-impeded mechanism analysis as follows [S15, S16]:

At a steady state, Donnan equilibrium is established between the hydrogel evaporator and the brine:

$$c^b_{Na^+} c^b_{Cl^-} = c^h_{Na^+} c^h_{Cl^-} \tag{S7}$$

where $c^b_{Na^+}$ and $c^b_{Cl^-}$ are the concentrations of Na^+ and Cl^- in the brine, and $c^h_{Na^+}$ and $c^h_{Cl^-}$ are the concentrations of Na^+ and Cl^- in the hydrogel evaporator. According to the electroneutrality theory:

$$c^h_{Na^+} = c^h_{R^-} + c^h_{Cl^-} \tag{S8}$$

$$c^b_{Na^+} = c^b_{Cl^-} \tag{S9}$$

where $c^h_{R^-}$ represents the concentration of the fixed sulfonic acid groups. Combining the above equations, we obtain:

$$\left(c^b_{Cl^-}\right)^2 = \left(c^h_{Cl^-}\right)^2 + c^h_{R^-} c^h_{Cl^-} \tag{S10}$$

Thus, the concentration of Cl^- entering the hydrogel evaporator is inversely proportional to that of fixed sulfonic acid groups, expressed as follows:

$$c^h_{R^-} = \frac{\left(c^b_{Cl^-}\right)^2}{c^h_{Cl^-}} - c^h_{Cl^-} \tag{S11}$$

Therefore, the introduction of sulfonic acid groups ($c^h_{R^-}$) impedes the anions ($c^h_{Cl^-}$) from entering the evaporator. In addition, according to Eq. (9), the brine should maintain electroneutrality, thus the Na^+ ($c^h_{Na^+}$) entering the evaporator is impeded.

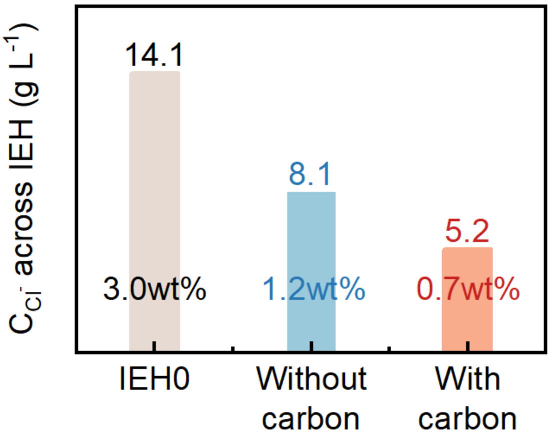

Fig. S18 Concentration of chloride ions permeated across IEHs in 20 wt% brine after 8 h

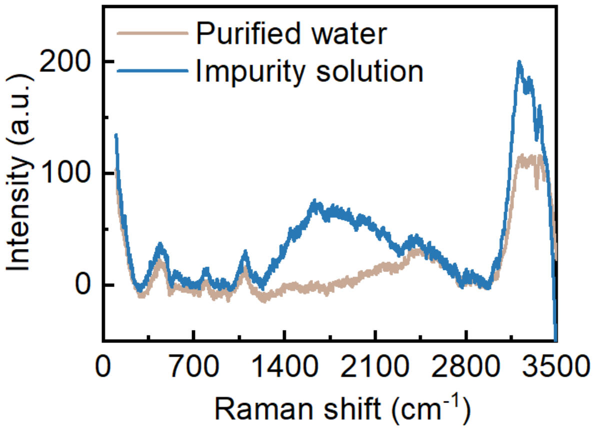

Fig. S19 Raman spectra of the waste washes with negative charged surfactant impurities before and after purification

S1.14 Calculation of Energy Efficiency based on IEHs

The evaporation rates were calculated using the projected area. There are two reasons for this: The energy input on the top projected surface is equal to the total energy input of the evaporator; During the evaporation process, shading treatment was carried out by wrapping foam around the container, preventing additional energy input. In addition, when the system under illumination reaches a steady-state, the absorber's temperature is 31.2 °C, which is much higher than 25 °C of environment. So, we didn't take the dark evaporation rate in the calculation [S17].

The efficiency of solar-to-vapor conversion (η) was expressed as follows [17]:

$$\eta = \frac{\dot{m} E_{ec}}{U_{in}} \tag{S12}$$

where $\dot{m}$ is the evaporation rate based on IEHs (Fig. 3h). Each value should be calculated based on at least three parallel measurements under the same conditions.

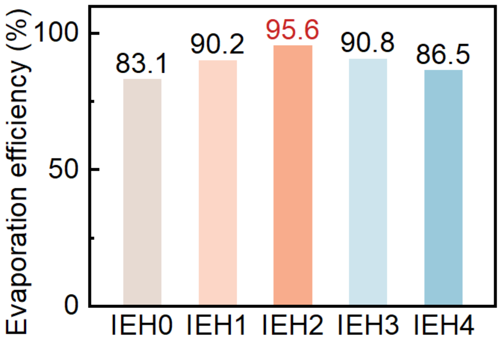

Fig. S20 Calculated evaporation efficiency of IEHs

S1.15 Evaporation Enthalpy and Performance with Changing Salt Ion Concentration

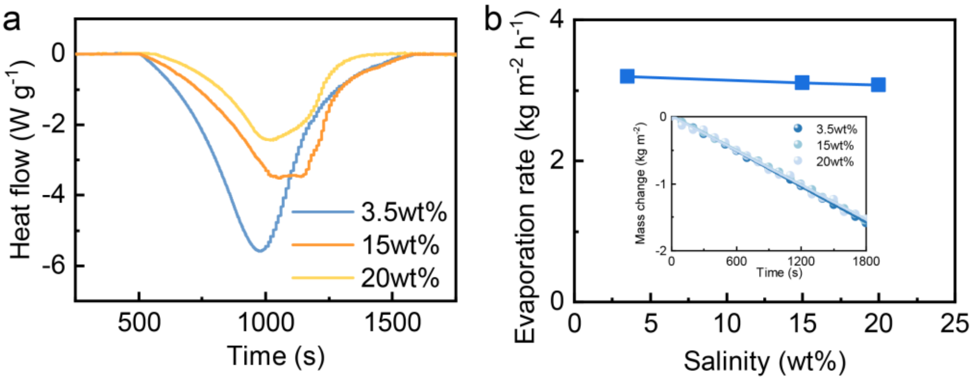

Fig. S21 Enthalpy of evaporation (**a**) and evaporation performance (**b**) of the IEH2 in 3.5wt%, 15wt%, and 20wt% brine

S1.16 Temperature of the Evaporation Surface of IEH0

The stable temperature is 33.51 °C. The low temperature is contributed by the high-efficiency evaporation. In fact, the temperatures of the most hydrogel-based evaporators are lower than other evaporators [S18, S19].

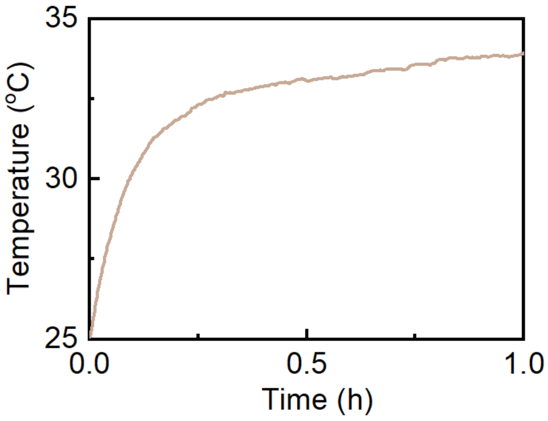

Fig. S22 Temperature of the photothermal evaporation surface of IEH0

S1.17 Mechanical Properties of IEH2 after 15-day Desalination

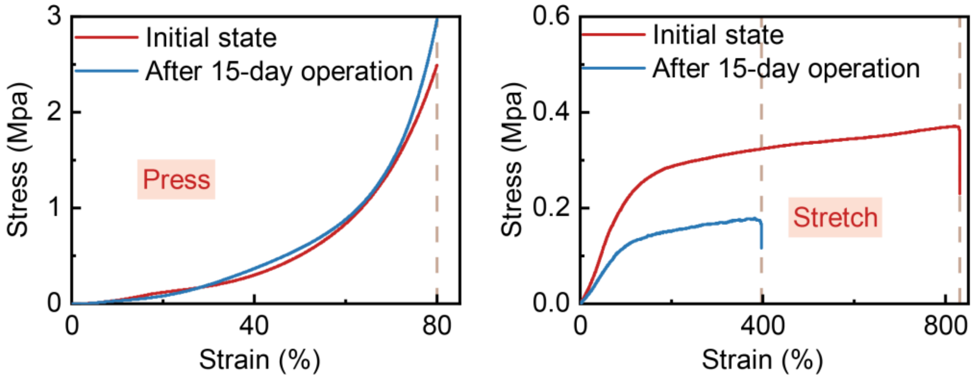

Fig. S23 Mechanical properties before and after the 15-day continuous operation in 20wt% brine

S1.18 Comparison of the Stability among Different IEHs

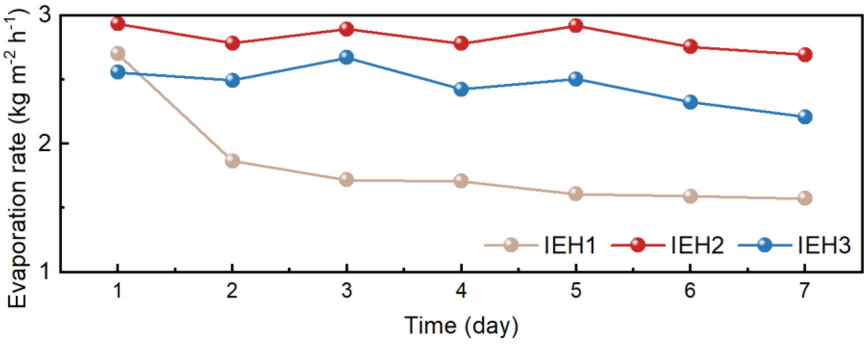

Fig. S24 Stability data of IEH1, IEH2, and IEH3

S1.19 Comparison of IEH2 with Other Evaporators

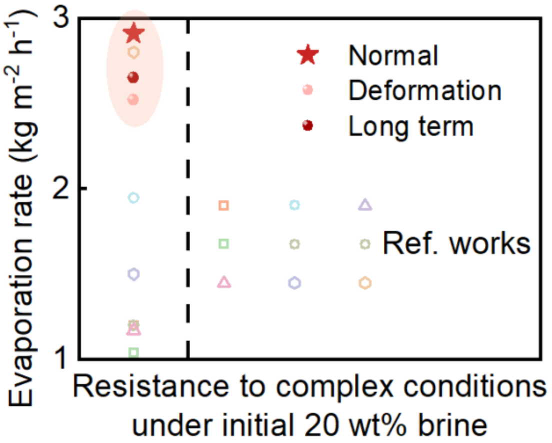

Fig. S25 Comparison in evaporation rates in 20 wt% brine of the IEH2 under complex conditions and other reported literature under normal conditions

S1.20 Electricity Generation Procedure and Performance

An Ag electrode (1×37 mm, Shanghai Yueci Electronic Technology co., LTD) and a custom-made AgCl electrode were used for recording electricity. Before the experiment, the IEH was assembled with a customized quartz device and sealed well. The specific experiment rules are that the 25 wt% solution and the 0.025 or 3.5 wt% solution were placed on different sides of the IEH, respectively. Then, the mentioned electrodes were inserted into the solutions separately and ensured they were in good contact with the solutions, using alligator clips to

connect electrodes and the multimeter to monitor current and voltage outputs. After the whole device was connected completely, the startup time of the multimeter was directly related to the initial displayed value, thus we only focused on the absence or presence of signals and the corresponding change trend, demonstrating the salinity-gradient electricity generation. Note that there was no need to consider the positive and negative of the connection joint, and it only affected the symbol of the displayed numbers.

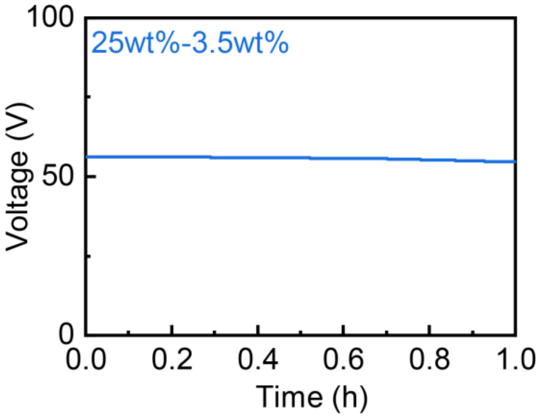

Fig. S26 Open-circuit voltage of the IEH2 under the salinity difference between the 25wt% and 3.5wt% NaCl solutions

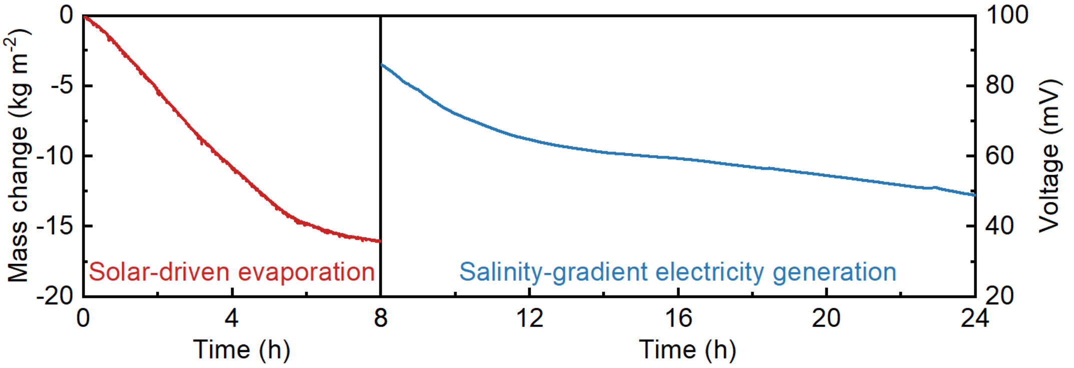

Fig. S27 *In-suit* outdoor experiment of solar-driven evaporation and salinity gradient electricity generation during the whole day. The simulated seawater with 3.5wt% salinity is used for the low salinity solution combined with the waste evaporated brine for generating electricity.

S1.21 Cost and Scalability of the IEH

(1) The cost of this material outperforms most of reported materials [S3, S4, S20-S31] Referring to the previous paper [S22], we normalize the cost-effectiveness(ε) as the defined to be evaporation rate (kg m^{-2} h^{-1}) per cost (\$ m^{-2}), which can be understood as how many grams of purified water can be received in one hour if spend one U.S. dollar.

Table S3 Detailed prices of raw materials and the total IEH evaporator

Raw materials	Cost (\$ m^{-2})	Cost-effectiveness (kg h^{-1} \$$^{-1}$)
AAM	0.29	
NaAMPS	0.28	
MBAA	0.00	
UV-I	0.02	
AG	0.85	
CB	0.01	
SDBS (45 ml solution)	0.13	
Total	1.58	**1.84**

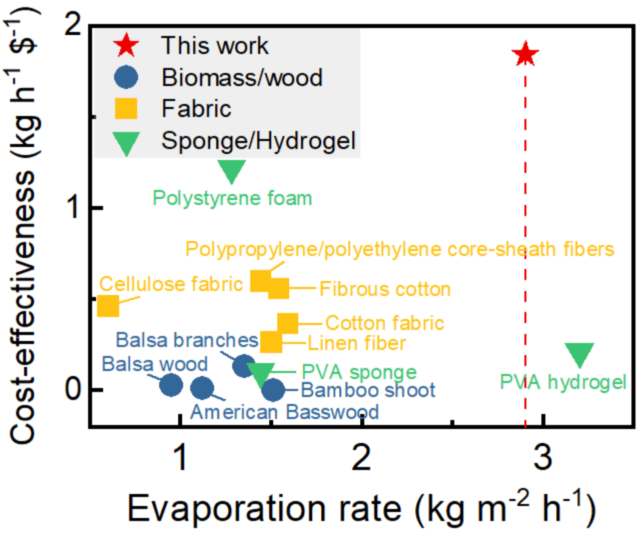

Fig. S28 Cost effectiveness comparison of the IEH with other reported evaporators

(2) The scalability of this material outperforms most of reported materials. Biomass-based/wood-based and other material-based solar evaporators are often limited by the size of raw materials, such as wood. In contrast, hydrogels provide enhanced scalability as their production involves pouring raw solutions into a mold for cross-linking, solely dependent on the mold size. This capability allows manufacturing in a wide range of sizes, spanning from very small to very large, which as shown in Fig. S29. Furthermore, we increase the size of the evaporator to 100 cm^2 for outdoor experiments with 0.9 sun irradiation, the evaporator still maintains a high evaporation rate of 2.5 kg m^{-2} h^{-1} (Fig. S30).

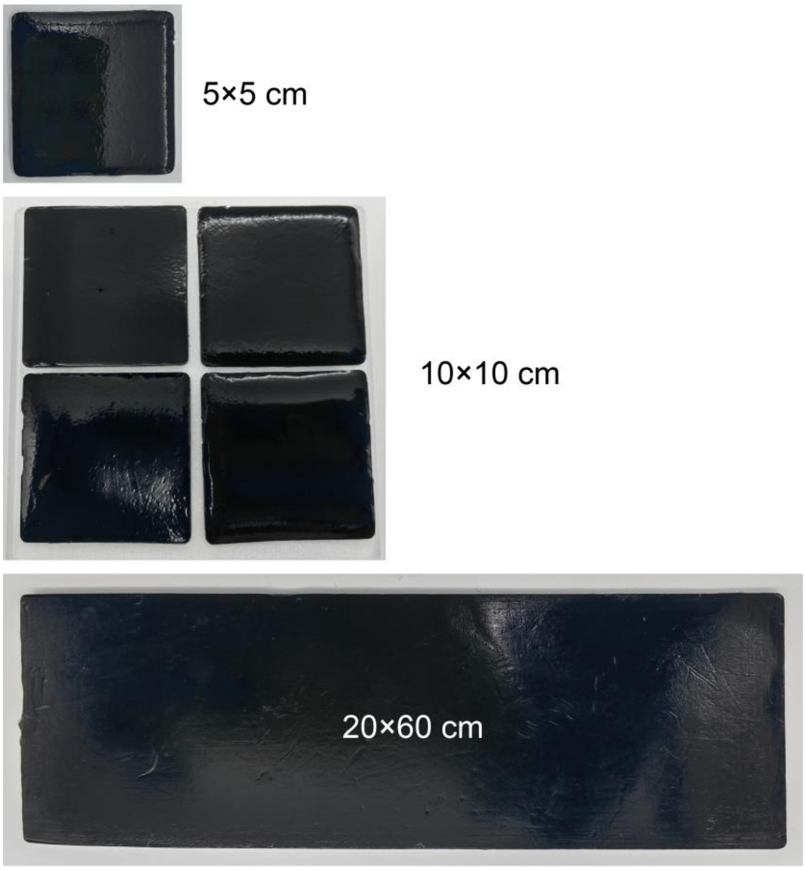

Fig. S29 Scalability of the IEH with difference sizes

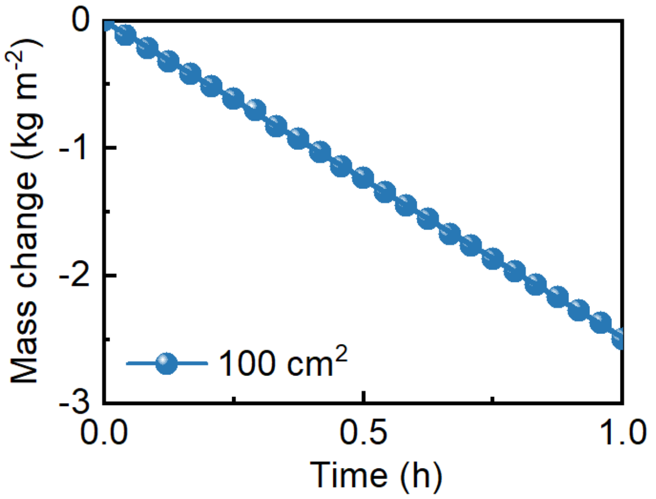

Fig. S30 Outdoor solar steam generation with a 100-cm^2 hydrogel evaporator

S2 Supplementary References

[S1] M. Gao, L. Zhu, C. K. Peh, G. W. Ho. Solar absorber material and system designs for photothermal water vaporization towards clean water and energy production. Energy Environ. Sci. **12**(3), 841 (2019). https://doi.org/10.1039/c8ee01146j

[S2] C. Chen, Y. Kuang, L. Hu. Challenges and opportunities for solar evaporation. Joule **3**(3), 683 (2019). https://doi.org/10.1016/j.joule.2018.12.023

[S3] L. Liang, Y. Dong, Y. Liu, X. Meng. Modification of polyurethane sponge based on the thiol-ene click reaction and its application for oil/water separation. Polymers **11**(12), 2072 (2019). https://doi.org/10.3390/polym11122072

[S4] B. Wenn, T. Junkers. Continuous microflow photoraft polymerization. Macromolecules **49**(18), 6888 (2016). https://doi.org/10.1021/acs.macromol.6b01534

[S5] F. Mo, Z. Chen, G. Liang, D. Wang, Y. Zhao, H. Li, B. Dong, C. Zhi. Zwitterionic sulfobetaine hydrogel electrolyte building separated positive/negative ion migration channels for aqueous Zn-MnO_2 batteries with superior rate capabilities. Adv. Energy Mater. **10**(16), 2000035 (2020). https://doi.org/10.1002/aenm.202000035

[S6] F. Zhao, X. Zhou, Y. Shi, X. Qian, M. Alexander, X. Zhao, S. Mendez, R. Yang, L. Qu, G. Yu. Highly efficient solar vapour generation via hierarchically nanostructured gels. Nat. Nanotechnol. **13**(6), 489 (2018). https://doi.org/10.1038/s41565-018-0097-z

[S7] Y. Guo, H. Lu, F. Zhao, X. Zhou, W. Shi, G. Yu. Architecting highly hydratable polymer networks to tune the water state for solar water purification. Sci. Adv. **5**(6), eaaw5484 (2019). https://doi.org/10.1126/sciadv.aaw5484

[S8] H. Zou, X. Meng, X. Zhao, J. Qiu. Hofmeister effect-enhanced hydration chemistry of hydrogel for high-efficiency solar-driven interfacial desalination. Adv. Mater. **35**(5), 2207262 (2023). https://doi.org/10.1002/adma.202207262

[S9] Z. Liu, Z. Zhou, N. Wu, R. Zhang, B. Zhu, H. Jin, Y. Zhang, M. Zhu, Z. Chen. Hierarchical photothermal fabrics with low evaporation enthalpy as heliotropic evaporators for efficient, continuous, salt-free desalination. ACS Nano **15**(8), 13007 (2021). https://doi.org/10.1021/acsnano.1c01900

[S10] P. Demianenko, B. Minisini, M. Lamrani, F. Poncin-Epaillard. How the structural and physicochemical properties of polyacrylamide/alginate hydrogel influence its oxygen

permeability. Polym. Test **53**, 299 (2016). https://doi.org/10.1016/j.polymertesting.2016.06.015

[S11] C. Qiao, X. Ma, J. Zhang, J. Yao. Effect of hydration on water state, glass transition dynamics and crystalline structure in chitosan films. Carbohydr. Polym. **206**, 602 (2019). https://doi.org/10.1016/j.carbpol.2018.11.045

[S12] X. Zhou, F. Zhao, Y. Guo, Y. Zhang, G. Yua. A hydrogel-based antifouling solar evaporator for highly efficient water desalination. Energy Environ. Sci. **11**, 1985 (2018). https://doi.org/10.1039/x0xx00000x

[S13] M. H. Sharqawy, J. H. Lienhard, S. M. Zubair. Thermophysical properties of seawater: A review of existing correlations and data. Desalination Water Treat. **16**(1-3), 354 (2012). https://doi.org/10.5004/dwt.2010.1079

[S14] J. Hopfner, C. Klein, M. Wilhelm. A novel approach for the desalination of seawater by means of reusable poly(acrylic acid) hydrogels and mechanical force. Macromol Rapid Commun. **31**(15), 1337 (2010). https://doi.org/10.1002/marc.201000058

[S15] Sudipta Sarkar, Arup K. SenGupta, P. Prakash. The donnan membrane principle: Opportunities for sustainable engineered processes and materials. Environ. Sci. Technol. **44**(4), 1161 (2010). https://doi.org/10.1021/es9024029

[S16] K. Zuo, K. Wang, R. M. DuChanois, Q. Fang, E. M. Deemer, X. Huang, R. Xin, I. A. Said, Z. He, Y. Feng, W. Shane Walker, J. Lou, M. Elimelech, X. Huang, Q. Li. Selective membranes in water and wastewater treatment: Role of advanced materials. Mater. Today **50**, 516 (2021). https://doi.org/10.1016/j.mattod.2021.06.013

[S17] X. Li, G. Ni, T. Cooper, N. Xu, J. Li, L. Zhou, X. Hu, B. Zhu, P. Yao, J. Zhu. Measuring conversion efficiency of solar vapor generation. Joule **3**(8), 1798 (2019). https://doi.org/10.1016/j.joule.2019.06.009

[S18] Y. Guo, X. Zhou, F. Zhao, J. Bae, B. Rosenberger, G. Yu. Synergistic energy nanoconfinement and water activation in hydrogels for efficient solar water desalination. ACS Nano **13**(7), 7913 (2019). https://doi.org/10.1021/acsnano.9b02301

[S19] Y. Guo, C. M. Dundas, X. Zhou, K. P. Johnston, G. Yu. Molecular engineering of hydrogels for rapid water disinfection and sustainable solar vapor generation. Adv. Mater. **33**(35), 2102994 (2021). https://doi.org/10.1002/adma.202102994

[S20] L. Song, P. Mu, L. Geng, Q. Wang, J. Li. A novel salt-rejecting linen fabric-based solar evaporator for stable and efficient water desalination under highly saline water. ACS Sustain. Chem. Eng. **8**(31), 11845 (2020). https://doi.org/10.1021/acssuschemeng.0c04407

[S21] Y. Zhu, G. Tian, Y. Liu, H. Li, P. Zhang, L. Zhan, R. Gao, C. Huang. Low-cost, unsinkable, and highly efficient solar evaporators based on coating mwcnts on nonwovens with unidirectional water-transfer. Adv. Sci. **8**(19), e2101727 (2021). https://doi.org/10.1002/advs.202101727

[S22] Y. Guo, H. Lu, F. Zhao, X. Zhou, W. Shi, G. Yu. Biomass-derived hybrid hydrogel evaporators for cost-effective solar water purification. Adv. Mater. **32**(11), 1907061 (2020). https://doi.org/10.1002/adma.201907061

[S23] M. Zhu, Y. Li, G. Chen, F. Jiang, Z. Yang, X. Luo, Y. Wang, S. D. Lacey, J. Dai, C. Wang, C. Jia, J. Wan, Y. Yao, A. Gong, B. Yang, Z. Yu, S. Das, L. Hu. Tree-inspired design for high-efficiency water extraction. Adv. Mater. **29**(44), 1704107 (2017). https://doi.org/10.1002/adma.201704107

[S24] S. Cheng, Z. Yu, Z. Lin, L. Li, Y. Li, Z. Mao. A lotus leaf like vertical hierarchical solar vapor generator for stable and efficient evaporation of high-salinity brine. Chem. Eng. J. **401**(1), 126108 (2020). https://doi.org/10.1016/j.cej.2020.126108

[S25] Y. Chen, J. Fang, T. Ling, M. Xia, P. Xu, Y. Cao, D. Wei, J. Gao. Bamboo shoot-based evaporator with self-cleaning and mildew-resistant for efficient solar steam generation. Desalination **541**, 116003 (2022). https://doi.org/10.1016/j.desal.2022.116003

[S26] H. Kou, Z. Liu, B. Zhu, D. K. Macharia, S. Ahmed, B. Wu, M. Zhu, X. Liu, Z. Chen. Recyclable cnt-coupled cotton fabrics for low-cost and efficient desalination of seawater under sunlight. Desalination **462**, 29 (2019). https://doi.org/10.1016/j.desal.2019.04.005

[S27] C. Dang, H. Wang, Y. Cao, J. Shen, J. Zhang, L. Lv, G. Xu, M. Zhu. Ultra salt-resistant solar desalination system via large-scale easy assembly of microstructural units. Energy Environ Sci. **15**(12), 5405 (2022). https://doi.org/10.1039/d2ee03341k

[S28] G. Ni, S. H. Zandavi, S. M. Javid, S. V. Boriskina, T. A. Cooper, G. Chen. A salt-rejecting floating solar still for low-cost desalination. Energy Environ. Sci. **11**(6), 1510 (2018). https://doi.org/10.1039/c8ee00220g

[S29] P. Xiao, J. Gu, C. Zhang, F. Ni, Y. Liang, J. He, L. Zhang, J. Ouyang, S.-W. Kuo, T. Chen. A scalable, low-cost and robust photo-thermal fabric with tunable and programmable 2d/3d structures towards environmentally adaptable liquid/solid-medium water extraction. Nano Energy **65**, 104002 (2019). https://doi.org/10.1016/j.nanoen.2019.104002

[S30] M. Yin, Y. Hsin, X. Guo, R. Zhang, X. Huang, X. Zhang. Facile and low-cost ceramic fiber-based carbon-carbon composite for solar evaporation. Sci. Total Environ. **759**, 143546 (2021). https://doi.org/10.1016/j.scitotenv.2020.143546

[S31] Wendong Wang, Joshua Giltinan, Svetlana Zakharchenko, M. Sitti. Dynamic and programmable self-assembly of micro-rafts at the air-water interface. Sci. Adv. **3**(5), e1602522 (2017). https://doi.org/10.1126/sciadv.1602522
